# Evidence for an enolate mechanism in the asymmetric Michael reaction of α,β-unsaturated aldehydes and ketones *via* a hybrid system of two secondary amine catalysts†

**DOI:** 10.1039/d0sc03359f

**Published:** 2020-09-21

**Authors:** Nariyoshi Umekubo, Takahiro Terunuma, Eunsang Kwon, Yujiro Hayashi

**Affiliations:** Department of Chemistry, Graduate School of Science, Tohoku University 6-3 Aramaki Aza Aoba, Aoba-ku Sendai 980-8578 Japan yujiro.hayashi.b7@tohoku.ac.jp; Research and Analytical Center for Giant Molecules, Graduate School of Science, Tohoku University Sendai 980-8578 Japan

## Abstract

The key nucleophile was found to be neither an enamine nor an enol, but an enolate in the direct Michael reaction of α,β-unsaturated aldehydes and non-activated ketones catalyzed by two amine catalysts namely diphenylprolinol silyl ether and pyrrolidine. This is a rare example of an enolate from a ketone serving as a key intermediate in the asymmetric organocatalytic reaction involving secondary amine catalysts because the ketone enolates are generally generated using a strong base, and the enamine is a common nucleophile in this type of reaction.

## Abstract 2

The key nucleophile was found to be neither an enamine nor an enol, but an enolate in the direct Michael reaction of α,β-unsaturated aldehydes and non-activated ketones catalyzed by two amine catalysts namely diphenylprolinol silyl ether and pyrrolidine.

## Graphical abstract

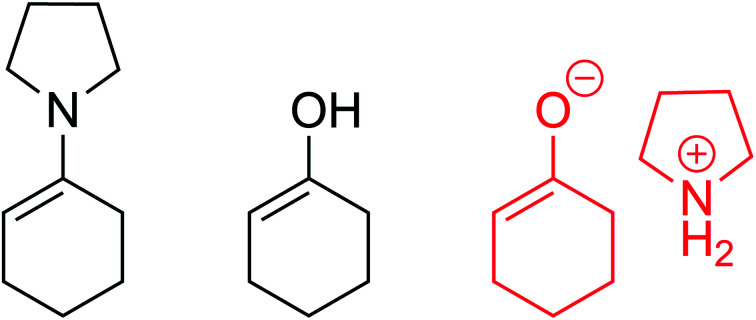

## Introduction

Enolates are versatile and important synthetic intermediates for the formation of α-substituted carbonyl compounds, which are widely used in the synthesis of organic molecules. In order to retard nucleophilic attack on the carbonyl group, and to match the weak acidity of the proton, sterically hindered strong amide bases such as lithium diisopropylamide (LDA) and lithium hexamethyldisilyl-amide (LHMDS) are generally employed for the generation of enolates from ketones.^1,2^ In the field of organocatalysis^3^ organic super bases such as phosphazene^4^ and proazaphosphatrane^5^ have been developed, which have similar or much stronger basicity than LDA and LHMDS.

Contrary to organic strong bases, weak secondary and primary amines can be successfully employed for the α-functionalization of non-activated ketones in which the key intermediate is not an enolate but an enamine. Stork was a pioneer in this field of enamine chemistry where an equimolar amount of the amine was employed.^6^ However, in recent asymmetric reactions, catalytically generated enamines are essentially the key intermediates. In this context, chiral secondary and primary amines act as catalysts for the α-functionalization of ketones in bond forming reactions such as the aldol reaction,^3,7^ Mannich reaction,^3,8^ Michael reaction,^3^ α-aminoxylation^3^ and α-aminations.^3^ In the Mannich reaction of acetone catalysed by a primary amine-thiourea organocatalyst, an enol mechanism was proposed,^9^ while an enamine mechanism is proved by calculation and ^13^C kinetic isotope effects in the Michael reaction of acetone catalysed by a similar catalyst.^10^ In the Michael reaction of propanal and methyl vinyl ketone catalysed by pyrrolidine, both enamine and enol mechanisms were computationally investigated, and an enamine is concluded to be a key nucleophile.^11^ The discrimination of the reaction mechanism of enamine and enol is difficult, and the asymmetric reaction involving an enol is rare. Moreover, the reaction is very rare in which an enolate (not enol) is a key intermediate in the secondary amine mediated asymmetric catalytic reactions of non-activated ketones with excellent enantioselectivity. Although there is one report involving an enolate as an intermediate, as far as we are aware, the enantioselectvitiy is not sufficient.^12^ In this study, we will demonstrate that the key nucleophile in the Michael reaction of cyclohexanone and α,β-unsaturated aldehyde catalyzed by a secondary amine was neither an enamine nor an enol but a rather unexpected enolate.

## Results and discussion

Recently, we reported the first direct asymmetric Michael reaction of non-activated ketones and α,β-unsaturated aldehydes catalyzed by a combination of two organocatalysts namely diphenylprolinol silyl ether **1**^13^ and pyrrolidine or 4-hydroxyproline (**2**) (eqn (1), Table 1, entries 1, 2 and 3).^14^ Although two similar pyrrolidine-type catalysts were involved in the reaction, an iminium ion generated from the α,β-unsaturated aldehyde was the reactive Michael acceptor based on Mayr's electrophilicity principle.^15^ This implies that the enantio face-selectivity of the α,β-unsaturated aldehyde was controlled by diphenylprolinol silyl ether. We hypothesized that the nucleophile would be an enamine generated from the reaction of the ketone and either pyrrolidine or 4-hydroxyproline. It has been well established that the enamine generated from the ketone and proline is a reactive nucleophile.^3,16^ We reasoned that if the second amine can control the enantio-face selectivity of the ketone, both *syn* and *anti*-isomers can be selectively synthesized.^17^ This hypothesis led us to study the effect of the second chiral amine catalyst in more detail with the intention of enhancing the selective control of relative stereochemistry.

**Table 1:** The effect of the amines in the asymmetric Michael reaction of diphenylprolinol silyl ether^a^

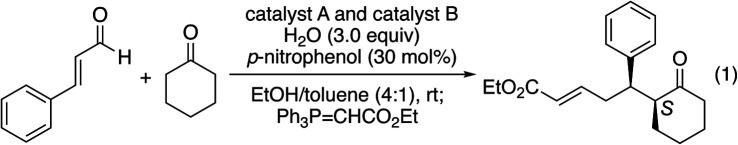						
Entry	Catalyst A	Catalyst B	Time [h]	dr^b^	Yield^c^ [%]	ee^d^ [%]
1	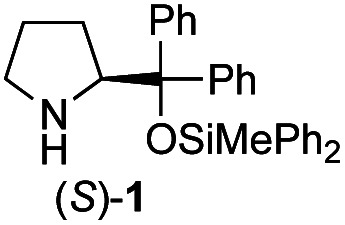	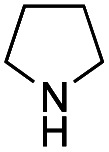	24	5 : 1	74	91
2	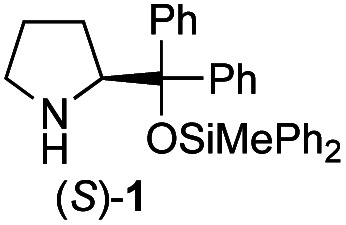	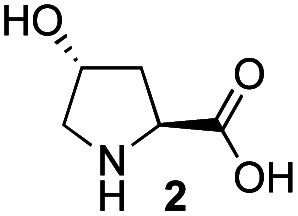	40	15 : 1	74	96
3	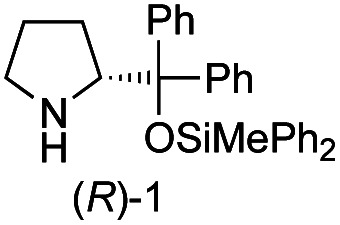	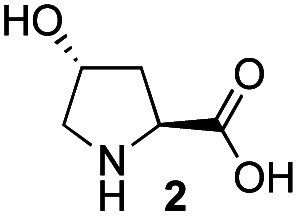	40	10 : 1	70	−95
4	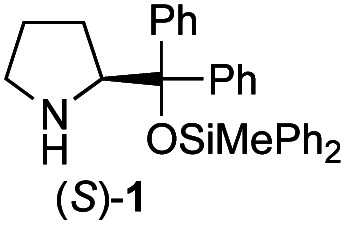	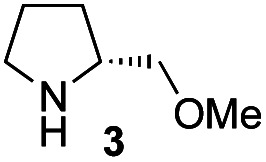	30	5 : 1	68	93
5	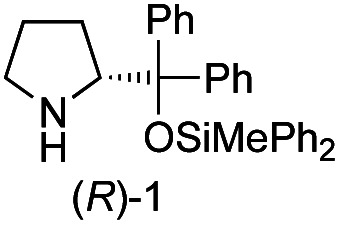	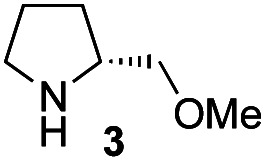	30	9 : 1	70	−94

a Unless otherwise shown, the reaction was performed by employing cinnamaldehyde (0.5 mmol), cyclohexanone (1.5 mmol), catalyst A (0.075 mmol), catalyst B (0.0375 mmol), *p*-nitrophenol (0.15 mmol), and water (1.5 mmol), in EtOH (0.4 mL) and toluene (0.1 mL) at room temperature. After the reaction, Wittig reagent (0.75 mmol) was added. See the ESI for details.

b dr ratio (*syn* : *anti*) was determined by ^1^H-NMR.

c Isolated yield of the diastereomer mixture.

d Determined by HPLC analysis on a chiral column material.

In our previous paper, we reported the results of (*S*)-diphenylprolinol silyl ether (*S*)-**1** with (2*S*,4*R*)-4-hydroxyproline **2** and (*R*)-**1** with (2*S*,4*R*)-**2** in the Michael reaction of cinnamaldehyde and cyclohexanone (Table 1, entries 2 and 3).^14^ Although the enantio face-selectivity of an enamine originating from (2*S*,4*R*)-4-hydroxyproline **2** is not known, that of an (*R*)-2-(methoxymethyl)pyrrolidine **3** has been well investigated.^18^ Thus, the effect of this amine **3** with a combination of (*S*)-**1** and (*R*)-**1** was examined (entries 4 and 5). Although the diastereoselectivity was slightly different according to the relative fractions of the two catalysts, the *syn*-isomer was predominantly obtained in all cases. As for the enantioselectivity, the absolute configuration was controlled by the chirality of the diphenylprolinol silyl ether **1** (ref. 19) regardless of the chirality of the second amine catalyst **3**. These results cast a doubt about the involvement of any enamine as an intermediate in the reaction.

**Fig. 1:**
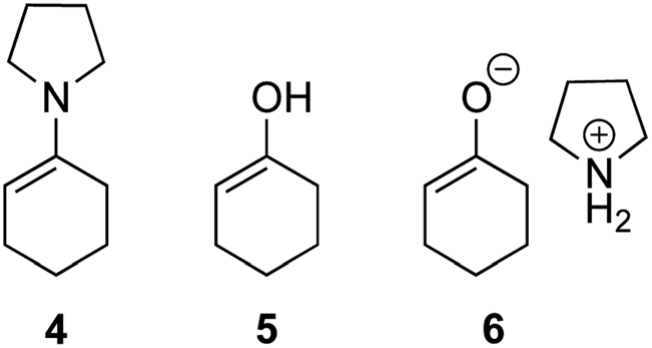
Enamine, enol and enolate.

**Scheme 1:**
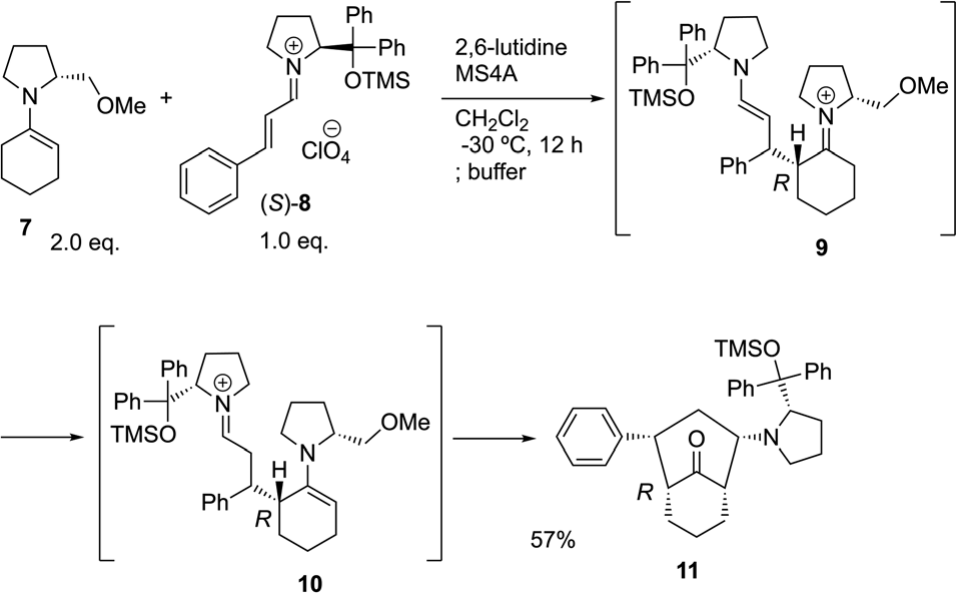
The reaction of enamine **7** and iminium ion (*S*)-**8**.

The possible nucleophiles would be the enamine **4**, the enol **5** and the enolate **6** (Fig. 1). Hence, we investigated the reactivity of these species. The reactivity of the enamine **7**,^18^ which was generated from (*R*)-2-(methoxymethyl)pyrrolidine **3**, was examined (Scheme 1). The equimolar reaction of enamine **7** and iminium ion (*S*)-**8** (ref. 20) proceeds in the presence of 2,6-lutidine and MS4A to afford the bicyclic compound **11** (ref. 21) in 57% yield. **11** was formed by the Michael reaction, followed by an exchange of the enamine and iminium ion and cyclization.^22^ The stereochemistry at the α-position of the obtained cyclohexanone was *R* whereas the observed stereochemistry from the catalytic Michael reaction was the opposite *S* (Table 1, entry 4).^23^ This result implies that the enamine **7** was not an intermediate in the catalytic reaction. Moreover, apart from the secondary amine, tertiary amines such as i-Pr_2_NEt can successfully act as a co-catalyst to afford the Michael product with excellent enantioselectivity (71%, *syn* : *anti* = 5 : 1, 97% ee). This result was also a piece of evidence to support the claim that the enamine was not a key nucleophile in the reaction.

The equimolar reactions of the iminium ion with 1-methoxycyclohex-1-ene and 1-(trimethylsiloxy)cyclohex-1-ene, and a combination of 1-(trimethylsiloxy)cyclohex-1-ene and tris(dimethylamino)sulfonium difluorotrimethylsilicate (TASF) were investigated. The two former nucleophiles would possess a similar reactivity to 1-hydroxycyclohex-1-ene **5**, but they did not react at all even after a longer reaction time (eqn (2)). In the reaction of a combination of silyl enol ether and TASF, which is known to generate an enolate,^24^ the Michael product was obtained at −30 °C after 1 h with a similar diastereo- and enantio-selectivity as observed in the catalytic reaction (eqn (3)). This result indicates that an enolate was the likely intermediate.2
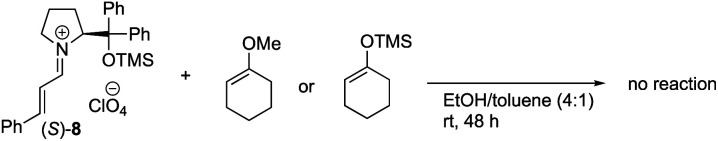
3
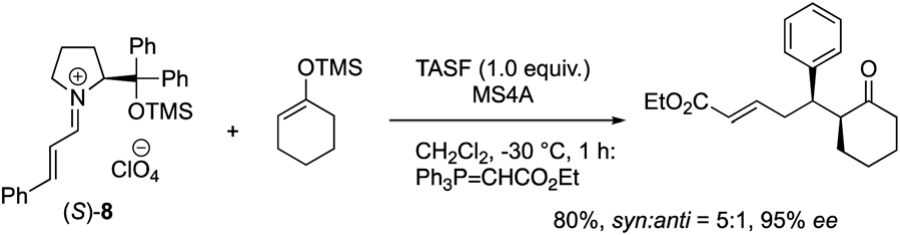


If the enolate was the actual nucleophile, a question arose about how the enolate would be generated from the ketone and pyrrolidine or i-Pr_2_NEt. The direct deprotonation of the ketone by pyrrolidine would be very difficult considering their relative p*K*_a_ values. That is to say that the p*K*_a_ value of an α-proton on cyclohexanone in DMSO is 26.4 (ref. 25) while that of an ammonium ion of Et_3_N is only 9.00.^26^ This is why a strong base such as LDA is usually employed. But the enolate could be generated considering an equilibrium between the keto and enol forms, and that the O–H proton of the enol form is rather acidic, although the content of enol is very low.^27^

Next, we investigated the generation speed of the enolate. The combined generation speed of both the enamine, the enol and the enolate can be monitored by the H/D exchange of the α-proton of the carbonyl group in the reaction with D_2_O (eqn (4)). The generation speed of the enamine can also be monitored by the ^16^O/^18^O exchange in the reaction with H_2_^18^O (eqn (5)).^28^ Moreover, as a tertiary amine can also acts as a co-catalyst in which the enamine would not be involved, the generation of only the enol and the enolate can be monitored by the H/D exchange reaction with D_2_O in the case of the tertiary amine (eqn (4)).4
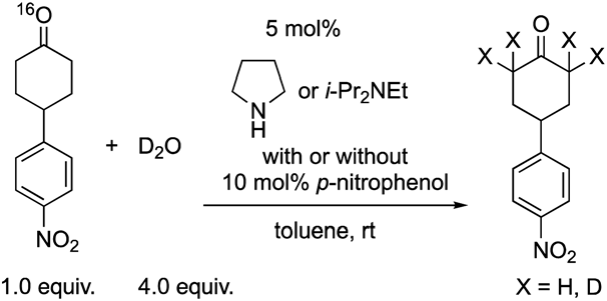
5
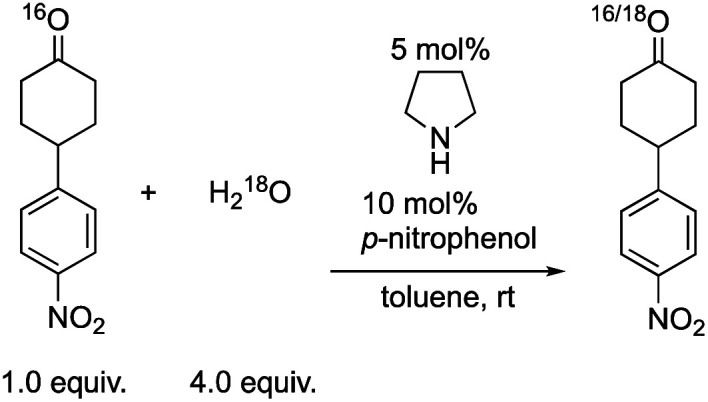


**Fig. 2:**
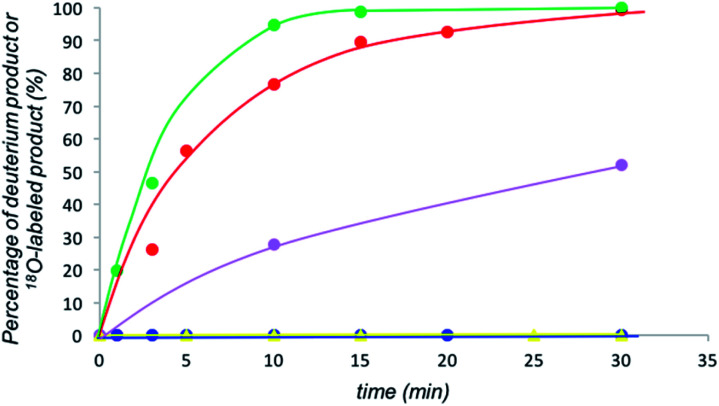
Generation of deuterated substrates in eqn (4), green: i-Pr_2_NEt and *p*-nitrophenol, red: pyrrolidine and *p*-nitrophenol, blue: *p*-nitrophenol, yellow: i-Pr_2_NEt, and generation of ^18^O labelled substrate in eqn (5), purple.

**Scheme 2:**
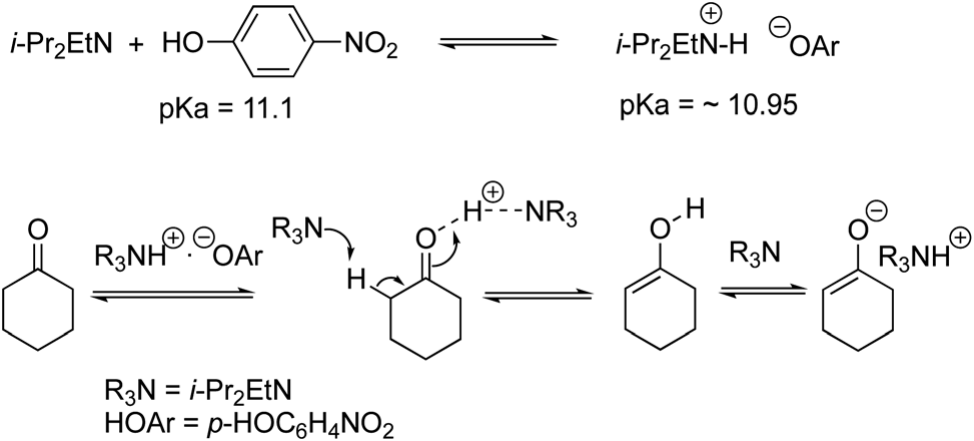
Generation of the enolate.

We examined the H/D exchange experiments in the presence of (1) pyrrolidine + *p*-nitrophenol (red), (2) *p*-nitrophenol (blue), (3) i-Pr_2_NEt + *p*-nitrophenol (green), and (4) i-Pr_2_NEt (yellow) (Fig. 2). A reaction using H_2_^18^O was conducted using pyrrolidine and *p*-nitrophenol (purple) (Fig. 2). Fig. 2 indicates that the generation speed of the enol and the enolate under the reaction conditions is rather fast compared to the Michael reaction. This indicates that the rapid kinetic of deprotonation is fast, although the concentration of the enol and enolate is quite low. The speed of generation of the enolate was faster in the case of i-Pr_2_NEt and *p*-nitrophenol compared to pyrrolidine and *p*-nitrophenol. Although each acid and base are known to accelerate the keto/enol equilibrium,^29^*p*-nitrophenol did not promote the H/D exchange under the reaction conditions. Moreover, the tertiary amine did not promote the exchange either. However, it should be noted that a combination of both acid and base facilitates the generation of the enolate. Their cooperative role would be explained as follows (Scheme 2):^30^*p*-nitrophenol and i-Pr_2_NEt partially form an ammonium ion, and all these species such as acids, bases and ammonium ions are present in a mixture under equilibrium based on their p*K*_a_ values.^26,31^ A protonation would occur at the carbonyl oxygen, which would increase the acidity of an α-proton of a carbonyl of cyclohexanone. Then, this α-proton of a carbonyl can be deprotonated by i-Pr_2_NEt. From the generated vinyl alcohol, deprotonation of the O–H proton^32^ would proceed very fast to afford the enolate.^33^ If so, the combination of acidity of the acid and basicity of the base should be important. In fact, when we used phenol or *p*-methoxyphenol^34^ instead of *p*-nitrophenol, the reaction became slow (see the ESI†). Moreover, the reaction did not proceed at all in the presence of CF_3_CO_2_H^35^ with a combination of i-Pr_2_NEt (see the ESI†).

One of the roles of the acid is to facilitate the generation of iminium ions from the α,β-unsaturated aldehyde and diphenylprolinol silyl ether, but this result indicates that another role of the acid is to accelerate the generation of the enolate in combination with the amine.

**Fig. 3:**
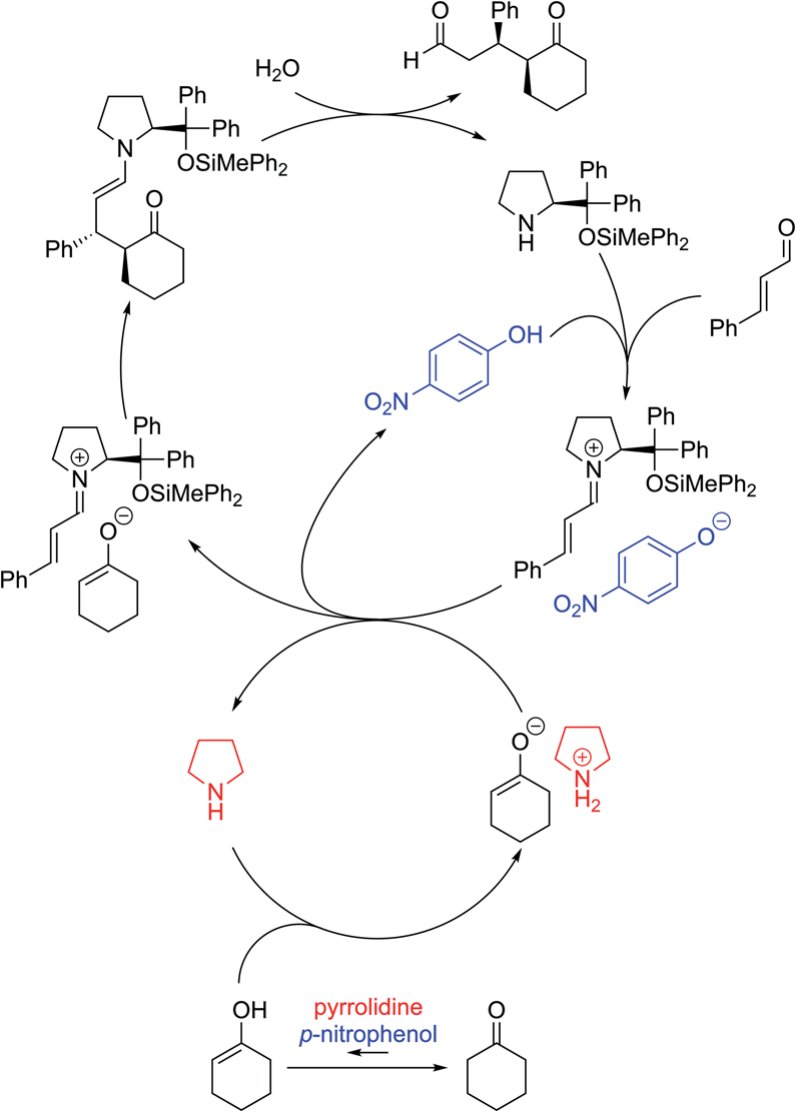
The catalytic cycle of the reaction.

Based on the above investigations, the reaction was considered to proceed as follows (Fig. 3): diphenylprolinol silyl ether reacts with cinnamaldehyde to generate an iminium salt. On the other hand, cyclohexanone is in equilibrium with the enol form in the presence of *p*-nitrophenol and pyrrolidine, and the enol tautomer would then react with another molecule of pyrrolidine to afford the ammonium enolate. Ion exchange occurs between the iminium salt and ammonium enolate, followed by a coupling reaction to provide the enamine, which is hydrolyzed to provide the Michael product with the regeneration of the catalyst. Thus, the role of the second amine was to accelerate the equilibrium of keto and enol with a combination of *p*-nitrophenol, and to also deprotonate the O–H proton in the enol tautomer of the cyclohexanone.

## Conclusions

In summary, we have identified the actual nucleophile in the direct Michael reaction of α,β-unsaturated aldehydes and non-activated ketones catalyzed by two amine catalysts. The generation speed of the enamine, enol, and enolate was examined along with the reactivity of these species using both catalytic and equimolar reactions of the isolated iminium ions (*S*)-**8** and (*R*)-**8**. The reaction was investigated using chiral (*R*)-2-(methoxymethyl)pyrrolidine **3** and its corresponding enamine from cyclohexanone with the chiral iminium ions (*S*)-**8** and (*R*)-**8**. We also investigated the reactivity of the enamine, the enol and the enolate ion. Based on these experiments, we have concluded that the key nucleophile in the direct Michael reaction was neither an enamine nor an enol, but an enolate. Although the enolate of cyclohexanone is usually generated with a strong base, a secondary amine can generate the enolate by the deprotonation of the O–H proton in the enol form. Even though the concentration of the enol form is very low, there is a rapid keto–enol conversion in the joint presence of an acid and a base compared with the Michael reaction. This is a rare asymmetric catalytic reaction using a secondary amine catalyst, in which the key nucleophile is not the enamine but the enolate of a non-activated ketone.

## Conflicts of interest

There are no conflicts to declare.

## Supplementary Material

SC-011-D0SC03359F-s001
