# Supplementary material for: Evidence for an enolate mechanism in the asymmetric Michael reaction of α,β-unsaturated aldehydes and ketones via a hybrid system of two secondary amine catalysts
Source: Chem Sci. 2020 Sep 21;11(41):11293–7. doi: 10.1039/d0sc03359f (PMC8162273; doi:10.1039/d0sc03359f)
Supplement: SC-011-D0SC03359F-s001 [file SC-011-D0SC03359F-s001.pdf]

Electronic Supplementary Material (ESI) for Chemical Science.
This journal is © The Royal Society of Chemistry 2020

Evidence for an enolate mechanism in the asymmetric Michael reaction of α,β-unsaturated aldehydes and ketones via hybrid system of two secondary amine catalysts

Nariyoshi Umekubo, Takahiro Terunuma, Eunsang Kwon, Yujiro Hayashi*

Department of Chemistry, Graduate School of Science, Tohoku University
6-3 Aramaki-Aza Aoba-ku, Sndai, Miyagi 980-8579, Japan

SUPPORTING INFORMATION

Experimental procedures and Characterization data

Table of Contents

1. Materials and Methods ……………………………………………………….……… Page S2
2. Experimental Procedures…………………………………………...…..… Page S2 – S6
   2. 1. Typical procedure of Michael reaction……………………………...… Page S2
   2. 2. The reaction between iminium ion and enamine ……………………….Page S3
   2. 3. The reaction between iminium ion and enol………………...……......Page S4
   2. 4. The reaction between iminium ion and enolate ...……………….......…Page S5
   2. 5. Study of generating speed of enamine and enol using $H_2{}^{18}O$ or D_2O …Page S5
   2. 6. Acid screening of Michael reaction……………….…………………...… Page S5
   2. 7. Compound information...………………......................................…Page S6-S7
3. References……………………………………………………………….……….Page S8

Spectra for Compounds…………………………………………....……... Page S9-S14
Mass Spectrum data…………………………………………………....…..... Page S15-S42
X-ray analysis data……………………………………...……......…... Page S43-S73

1. Materials and Methods

General Remarks: All reactions were carried out under argon atmosphere and monitored by thin-layer chromatography using Merck 60 F254 precoated silica gel plates (0.25 mm thickness). Specific optical rotations were measured using a JASCO P-1020 polarimeter and a JASCO DIP-370 polarimeter. FT-IR spectra were recorded on a JASCO FT/IR-410 spectrometer and a Perkin Elmer spectrum BX FT-IP spectrometer. 1H and 13C NMR spectra were recorded on an Agilent-400 MR (400 MHz for 1H NMR, 100 M Hz for 13C NMR) instrument. Data for ^{1}H NMR are reported as chemical shift (δ ppm), integration multiplicity (s = singlet, d = doublet, t = triplet, q = quartet, dd = doubledoublet, ddd = doubledoubledoublet, dt = doubletriplet, m = multiplet), coupling constant (Hz), Data for ^{13}C NMR are reported as chemical shift. High resolution ESI-TOF mass spectra were measured by Themo Orbi-trap instrument. HPLC analysis was performed on a HITACHI Elite LaChrom Series HPLC, UV detection monitored at appropriate wavelength respectively, using CHIRALPACK® ID (0.46 cm × 25 cm).

2. Experimental Procedures

2.1. Typical procedure of Michael reaction

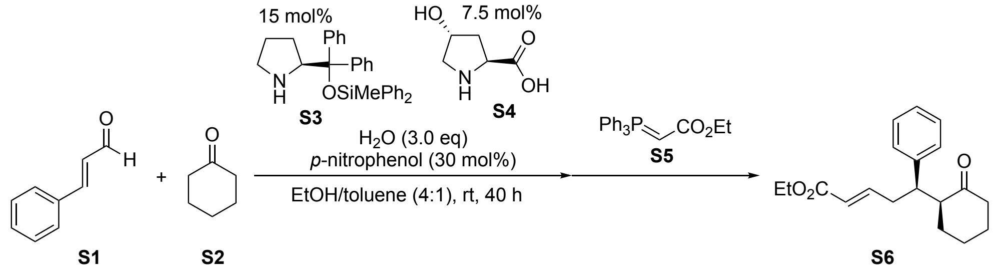

To a solution of cinnamaldehyde **S1** (66 mg, 0.5 mmol) and cyclohexanone **S2** (150 μl, 1.5 mmol) in EtOH/toluene=4:1 (500 μl), H_2O (27 μl, 1.5 mmol), catalyst **S3** (32 mg, 0.075 mmol), *p*-nitrophenol (21 mg, 0.15 mmol), hydroxyproline **S4** (4.9 mg, 0.0375 mmol) were added at room tempreture. After stirring the reaction mixture at this temperature for 40 h, Wittig reagent **S5** (266 mg, 0.75 mmol) was added. After stirring the reaction mixture at room temperature for 2 h, the reaction mixture was directly purified by column chromatography on silica gel (hexane:EtOAc = 12:1~10:1) to give the product[S1] **S6** (111 mg, 0.37 mmol) in 74% yield (*syn:anti*=15:1). The enantiomeric ratio was determined by HPLC using CHIRALPACK® ID (hexane/ ^{i}PrOH = 50:1; flow rate 1.0 ml/min, minor isomer t_R = 27.4 min, major isomer t_R = 29.6 min) (97% *ee*).

2.2. The reaction between iminium ion and enamine

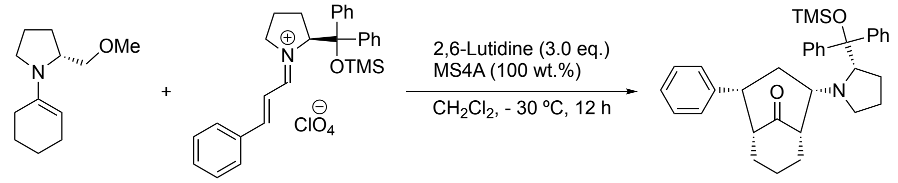

7 8 11

To a solution of enamine[S2] **7** (117 mg, 0.60 mmol) and 2,6-luthidine (104 µl, 0.90 mmol) in CH_2Cl_2 (2.0 ml), MS4A (162 mg, 100 wt.%), iminium salt [S3] **8** (162 mg, 0.30 mmol) were added at -30 ºC. After completion of reaction, the reaction mixture was quenched by the addition of phosphate buffer (2 mL) at room tempreture. The aqueous layer was extracted with EtOAc (5 mL) three times. The separated organic layers were dried over Na_2SO_4 and concentrated *in vacuo*. The crude material was purified by flash column chromatography on silica gel (EtOAc:hexane = 15:1) to afford the desired amine **11** in 57% yield (92.0 mg) as a yellow liquid.

To determine the structure of compound **11**, desilylation of compound **11** was peformed.

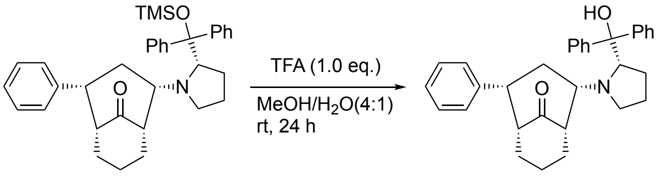

11 S7

To a solution of bicyclic compound **11** (80 mg, 0.15 mmol) in MeOH/H_2O=4:1 (2.0 ml), TFA (11.5 µl, 0.15 mmol) were added at room tempreture. After completion of reaction, the reaction mixture was quenched by the addition of aq. $NaHCO_3$ (2 mL) at room tempreture. The aqueous layer was extracted with EtOAc (5 mL) three times. The separated organic layers were dried over Na_2SO_4 and concentrated *in vacuo*. The crude material was purified by flash column chromatography on silica gel (EtOAc:hexane = 10:1) to afford the desired amine **S7** in 72% yield (50.3 mg) as a white solid. The structure of compound **S7** was determined by Xray crystal structure analysis.

2.3. The reaction between iminium ion and enol

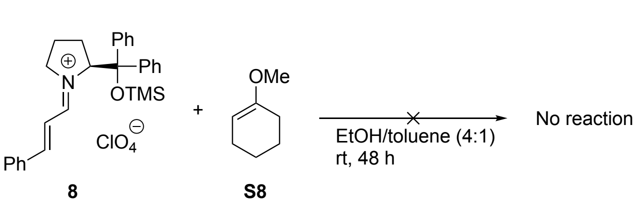

8 S8

To a solution of 1-methoxycyclohex-1-ene[S4] **S8** (39 mg, 0.30 mmol) in EtOH/toluene =

4:1 (2.0 ml), iminium salt[S3] **8** (162 mg, 0.30 mmol) were added at room tempreture. The reaction was monitored by TLC and NMR. No reaction was observed.

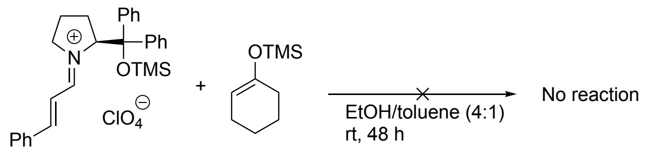

To a solution of (cyclohex-1-en-1-yloxy)trimethylsilane [S5] **S9** (39 mg, 0.30 mmol) in EtOH/toluene = 4:1 (2.0 ml), iminium salt[S2] **8** (162 mg, 0.30 mmol) were added at room tempreture. The reaction was monitored by TLC and NMR. No reaction was observed.

2.4. The reaction between iminium ion and enolate

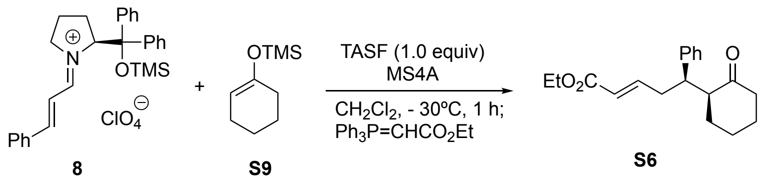

To a solution of (cyclohex-1-en-1-yloxy)trimethylsilane [S5] **S9** (39 mg, 0.30 mmol) and iminium salt[S4] **S8** (162 mg, 0.30 mmol) in CH_2Cl_2 (1.0 ml), TAFS (82.5 mg, 0.3 mmol) in CH_2Cl_2 (1.0 ml) was slowly added in 1 h by the use of syringe pump at -30 ºC. After completion of reaction, the reaction mixture was quenched by the addition of $Ph_3P{=}CHCO_2Et$ (160 mg, 0.45 mmol) at room tempreture. After stirring the reaction mixture at room temperature for 2 h, the reaction mixture was diluted by water (5 ml) and EtOAc (5 ml). The aqueous layer was extracted with EtOAc (5 mL) three times. The separated organic layers were dried over Na_2SO_4 and concentrated *in vacuo*. The crude material was purified by flash column chromatography on silica gel (EtOAc:hexane = 10:1) to afford the desired product S6[S1] in 65% yield (58.5 mg). The enantiomeric ratio was determined by HPLC using CHIRALPACK® ID (hexane/ ^{i}PrOH = 50:1; flow rate 1.0 ml/min, minor isomer t_R = 27.4 min, major isomer t_R = 29.6 min) (95% *ee*).

2.5. Study of generating speed of enamine and enol using $H_2{}^{18}O$ or D_2O

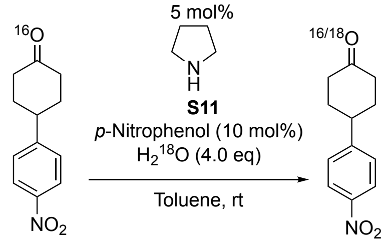

To a solution of ketone[S6] **S10** (72 mg, 0.33 mmol) in toluene (3.3 ml), pyrrolidine (1.36 μl, 0.0165 mmol), *p*-nitrophenol (4.59 mg, 0.033 mmol), $H_2{}^{18}O$ (26.4 μl, 1.32 mmol) were added at room temperature. The $^{16/18}O$ ratio of ketone **S12** was checked by MS spectroscopy at 10 min, 30 min, 45 min, 60 min, 90 min, and 120 min, respectively. The corresponding graph was illustrated as a purple line in Figure S1. These spectrums are shown in pages S18 ~ S23. The ^{16}O incorporation was determined by following calculation: 100- (Corrected ^{18}O ion insity) / (Corrected ^{18}O ion insity + Corrected ^{16}O ion insity) x 100 = XX% (^{18}O incorporation)

Typical procedure of study of generating speed of enamine and enol using D_2O

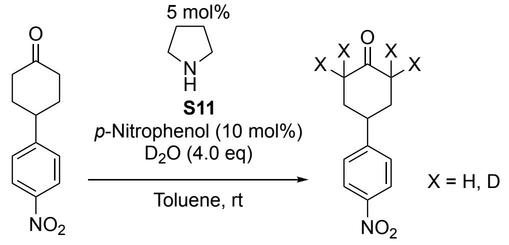

To a solution of ketone[S2] **S10** (72 mg, 0.33 mmol) in toluene (3.3 ml), pyrrolidine (1.36 μl, 0.0165 mmol)) and D_2O (26.4 μl, 1.32 mmol) were added at room temperature. After reduction of a part of ketone **S10** by $NaBH_4$ in MeOH, the amount of ketone **S13** was checked by MS spectroscopy at 1 min, 3 min, 5 min, 10 min, 15 min, 20 min, and 30 min respectively. The corresponding graph was illustrated as a red line in Figure S1. The incorporation of **S13** was determined by following calculation: 100-(Corrected ion insity of reduced **S13**) / (Corrected ion insity of reduced **S10**) x 100 = XX% (incorporation of **S17**)

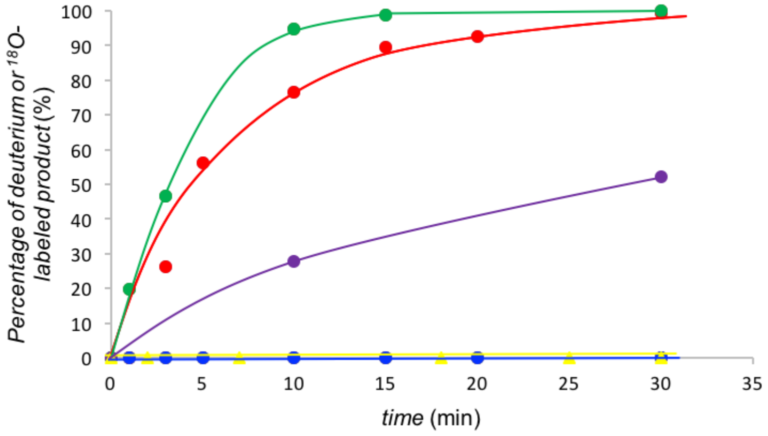

Green: $^{i}Pr_2NEt$ and *p*-nitrophenol, Red: pyrrolidine and p-nitrophenol, Purple: Generation of ^{18}O labelled substrate, Blue: *p*-nitrophenol, Yellow: i-Pr_2NEt.

2.6. Acid screening of Michael reaction

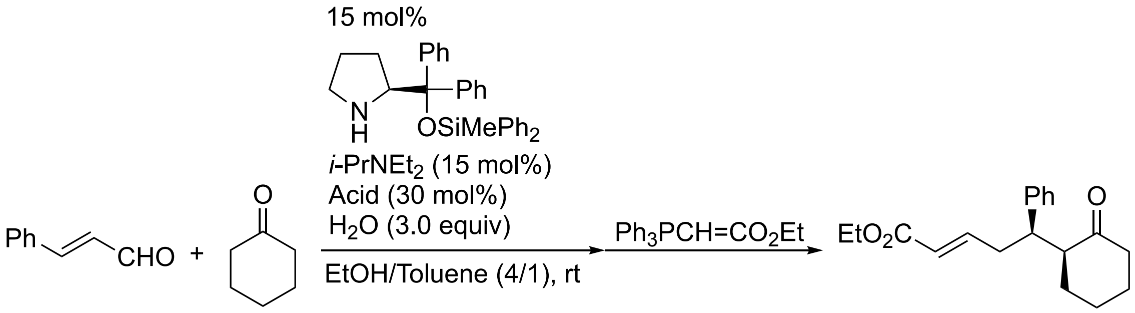

Entry	Acid	Time / h	Yield / %	*syn:anti*	Ee / %
1	*p*-nitophenol	24	71	5:1	97
2	*p*-methoxyphenol	96	72	6:1	n.d.
3	phenol	72	68	5:1	n.d.
4	TFA	96	no reaction	n.d.	n.d.

2.7. Compound information

(*1S*,*2S*,*4R*,*5R*)-2-((*S*)-2-(diphenyl((trimethylsilyl)oxy)methyl)pyrrolidin-1-yl)-4-phenylbicyclo[3.3.1]nonan-9-one (11)

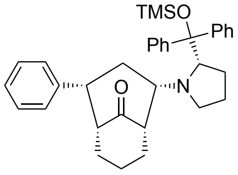

11

Physical state: yellow oil

^{1}H NMR ($CDCl_3$) δ -0.20 (s, 9H), 0.82 (m, 1H), 1.42 (m, 1H), 1.71 (m, 1H), 1.91 (m, 1H), 2.36 (m, 2H), 1.93 (m, 1H), 2.36 (m, 2H), 2.50 (m, 1H), 2.68 (dd, *J* = 3.6 Hz, 9.2 Hz, 1H), 2.90 (m, 2H), 3.07 (dd, *J* = 7.2 Hz, *J* = 19.2 Hz, 1H), 3.20 (d, *J* = 7.2 Hz, 1H), 4.22 (dd, *J* = 2.8 Hz, 9.8 Hz, 1H), 7.24 (m, 11H), 7.42 (m, 2H), 7.49 (m, 2H); ^{13}C NMR ($CDCl_3$) δ 21.1, 23.7, 28.4, 28.9, 29.4, 32.2, 44.8, 49.1, 49.6, 52.7, 61.7, 67.3, 85.2, 126.6, 121.7, 126.9 (2C), 127.0 (2C), 127.5 (2C), 127.8 (2C), 128.4 (2C), 129.4 (2C), 129.5 (2C), 129.6 (2C), 141.5, 1440, 144.2, 217.9; HRMS (ESI): $[M+H]^+$ calcd for $C_{35}H_{44}NO_2Si$: 538.3136, found: 538.3129; IR(neat)ν [cm^{-1}] 3450, 2937, 1709, 1644, 1494, 1447, 1249, 1206, 1157, 1067, 837, 701, 461, 448, 432.94 cm^{-1}, 408.83;$[\alpha]_D^{27}$ -57.2 (*c* 6.4, $CHCl_3$).

(1*S*,2*S*,4*R*,5*R*)-2-((*S*)-2-(hydroxydiphenylmethyl)pyrrolidin-1-yl)-4-phenylbicyclo [3.3.1]nonan-9-one (S7)

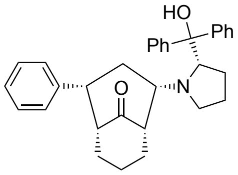

S7

Physical state: white solid (m.p. 148~151 ºC)

^{1}H NMR (*d*-benzene) δ 1.01 (m, 1H), 1.24 (m, 1H), 1.36 (m, 2H), 1.56 (m, 3H), 1.63 (m, 1H), 1.71 (m, 1H), 2.06 (m, 2H), 2.39 (m, 2H), 2.56 (dt, *J* = 9.2 Hz, *J* = 5.2 Hz, 1H), 2.66 (dd, *J* = 4.0 Hz, *J* = 8.8 Hz, 1H), 2.80 (m, 2H), 3.99 (dd, *J* = 4.2 Hz, *J* = 7.8 Hz, 1H), 6.77 (m, 3H), 7.05 (m, 8H), 7.65 (m, 4H); ^{13}C NMR ($CDCl_3$) δ21.1, 25.2, 28.2, 29.2, 30.2, 32.5, 44.5, 49.2, 51.5, 52.4, 62.1, 67.0, 78.0, 126.0, 126.3(2C), 126.7(2C), 127.8(2C),

128.0(2C), 128.1(2C), 128.2(2C), 128.5(2C), 141.4, 147.0, 149.0, 213.8; HRMS (ESI): $[M+H]^+$ calcd for $C_{32}H_{36}NO_2$: 466.6445, found: 466.6447; IR(neat)ν [cm^{-1}] 3452, 2930, 1713, 1644, 1494, 1449, 1273, 700, 414; $[\alpha]_D^{22}$ +18.2 (*c* 0.2, $CHCl_3$).

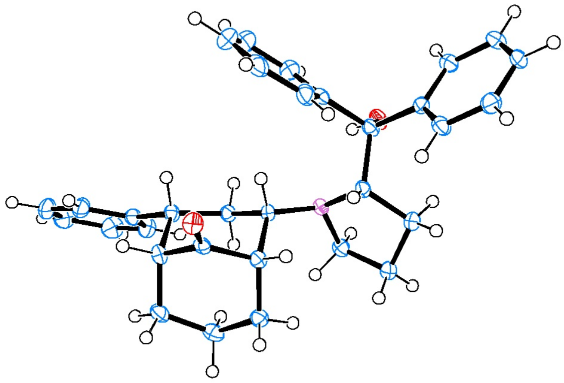

3. References

[S1] Y. Hayashi and N. Umekubo, *Angew. Chem. Int. Ed.*, 2018, **57**, 1958.

[S2] S. J. Blarer and D. Seebach, *Chem. Ber.* 1983, **116**, 2250.

[S3] H. Gotoh, T. Uchimaru and Y. Hayashi, *Chem. Eur. J.*, 2015, **21**, 12337.

[S4] E. Friedrich, H. O. Kalinowski and W. Lutz, *Tetrahedron*, 1980, **36**, 1051.

[S5] B. F. Marcune, S. Karady, P. J. Reider, R. A. Miller, M. Biba, L. DiMichele, and R. A. Reamer, *J. Org. Chem.*, 2003, **68**, 8088.

[S6] Y. Hayashi, T. Mukaiyama, M. Benohoud, N. R. Gupta, T. Ono, S. Toda, *Chem. Eur. J.*, 2016, **22**, 5868.

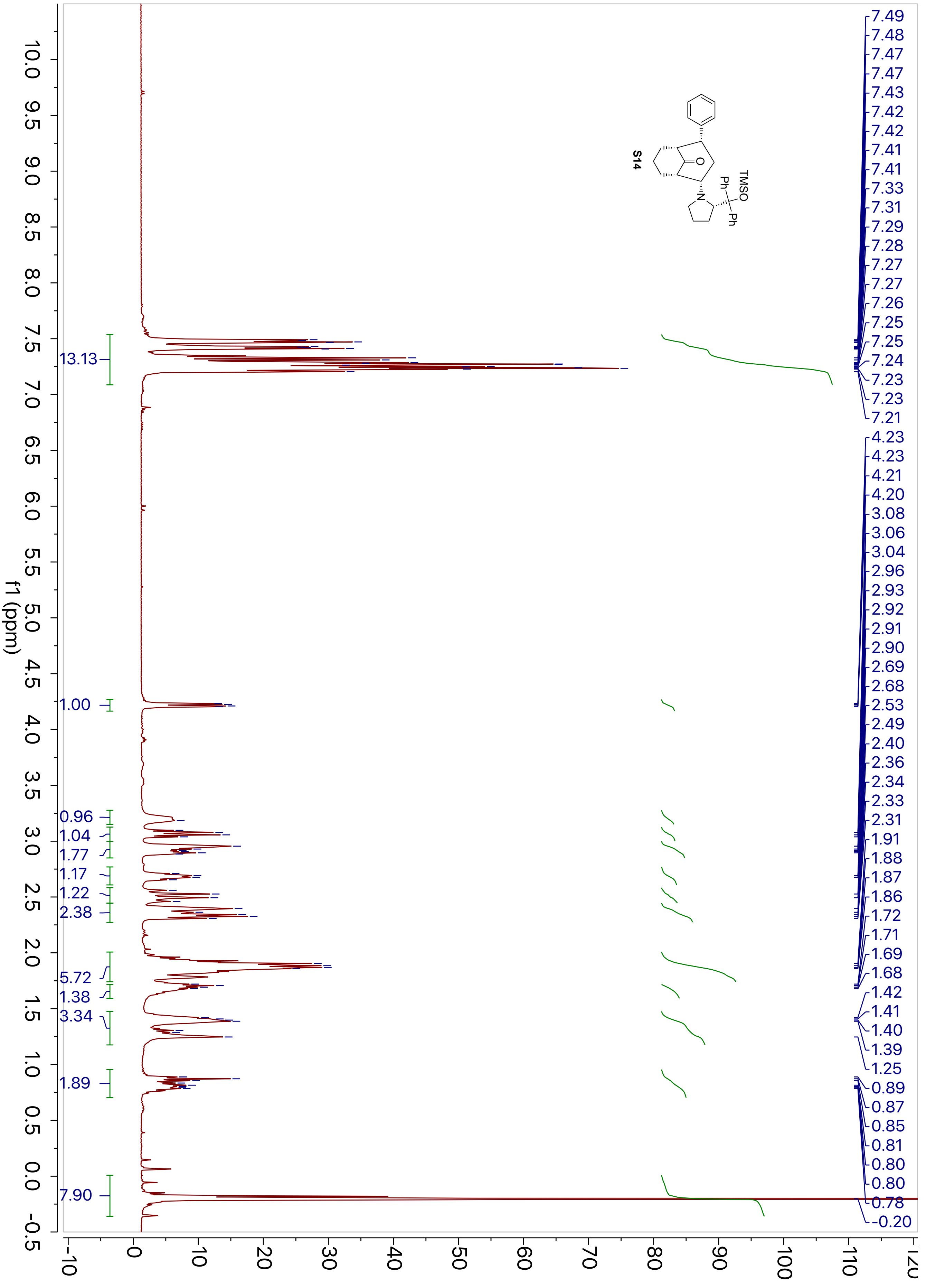
S14
TMSO
Ph
Ph
O
N
7.49
7.48
7.47
7.47
7.43
7.42
7.42
7.41
7.41
7.33
7.31
7.29
7.28
7.27
7.27
7.26
7.25
7.25
7.24
7.23
7.23
7.21
4.23
4.23
4.21
4.20
3.08
3.06
3.04
2.96
2.93
2.92
2.91
2.90
2.69
2.68
2.53
2.49
2.40
2.36
2.34
2.33
2.31
1.91
1.88
1.87
1.86
1.72
1.71
1.69
1.68
1.42
1.41
1.40
1.39
1.25
0.89
0.87
0.85
0.81
0.80
0.80
0.78
-0.20
13.13
1.00
0.96
1.04
1.77
1.17
1.22
2.38
5.72
1.38
3.34
1.89
7.90
f1 (ppm)

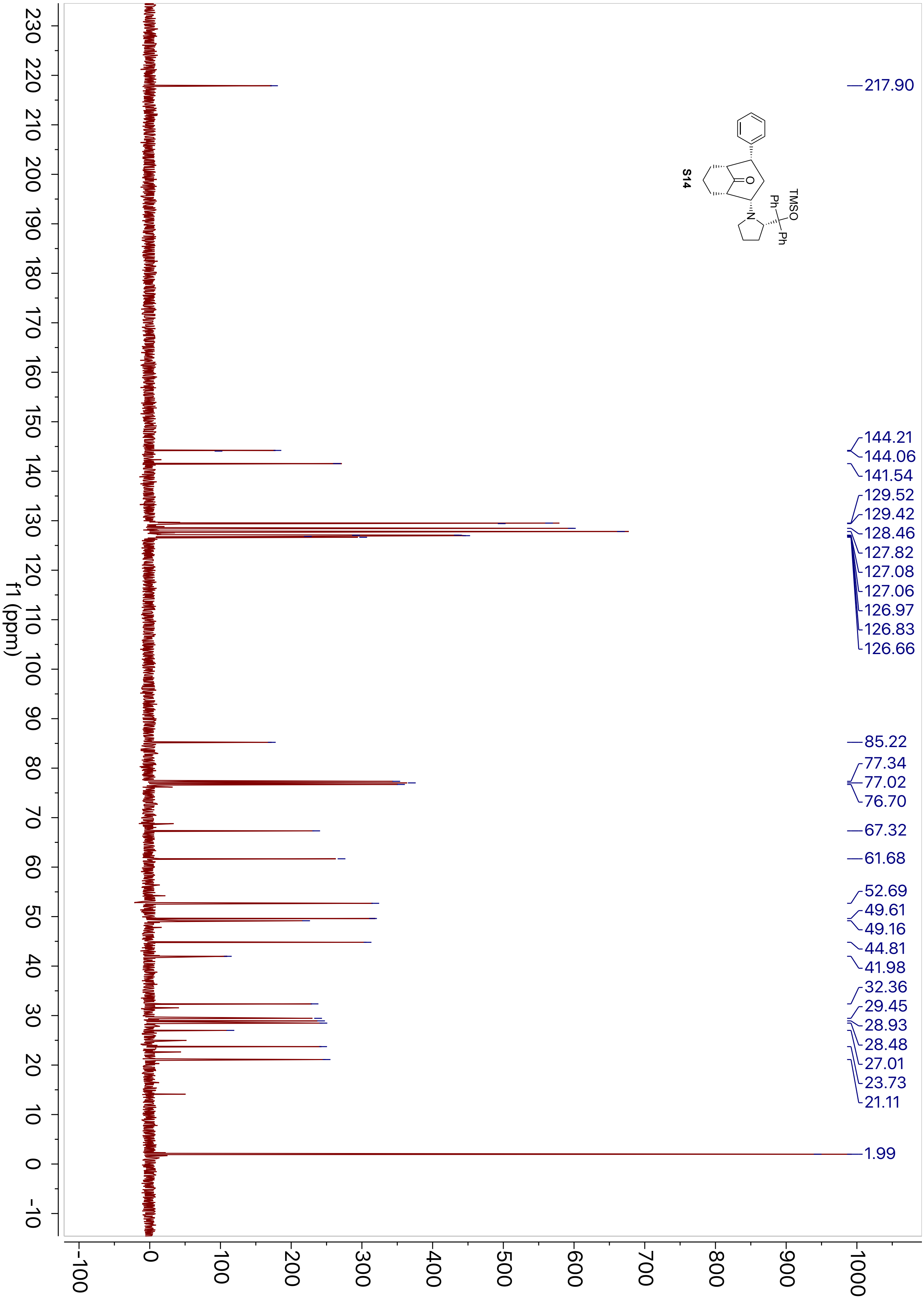
217.90
144.21
144.06
141.54
129.52
129.42
128.46
127.82
127.08
127.06
126.97
126.83
126.66
85.22
77.34
77.02
76.70
67.32
61.68
52.69
49.61
49.16
44.81
41.98
32.36
29.45
28.93
28.48
27.01
23.73
21.11
1.99
TMSO
Ph
Ph
O
N
S14
f1 (ppm)

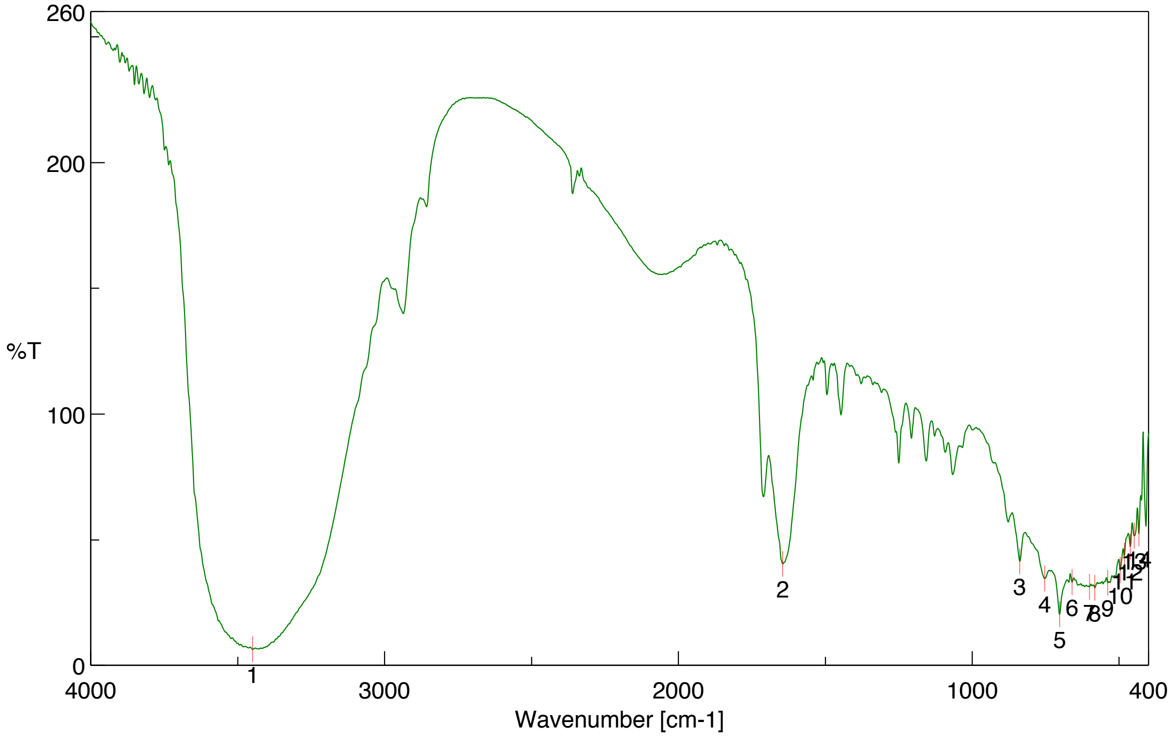

[ピーク検出結果] *

No.	位置 **	強度***	No.	位置**	強度 ***	No.	位置 **	強度***
1	3449.06	6.34454	2	1644.98	40.2916	3	837.919	41.3547
4	753.066	34.5128	5	701.962	20.3004	6	660.5	33.1265
7	600.717	31.2002	8	582.397	30.8904	9	539.007	32.8398
10	496.58	37.8842	11	482.117	43.6272	12	461.868	47.2416
13	448.369	51.5093	14	432.941	52.4477			

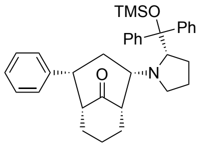

S14

* Detection result

** Wavenumber

*** Intensity

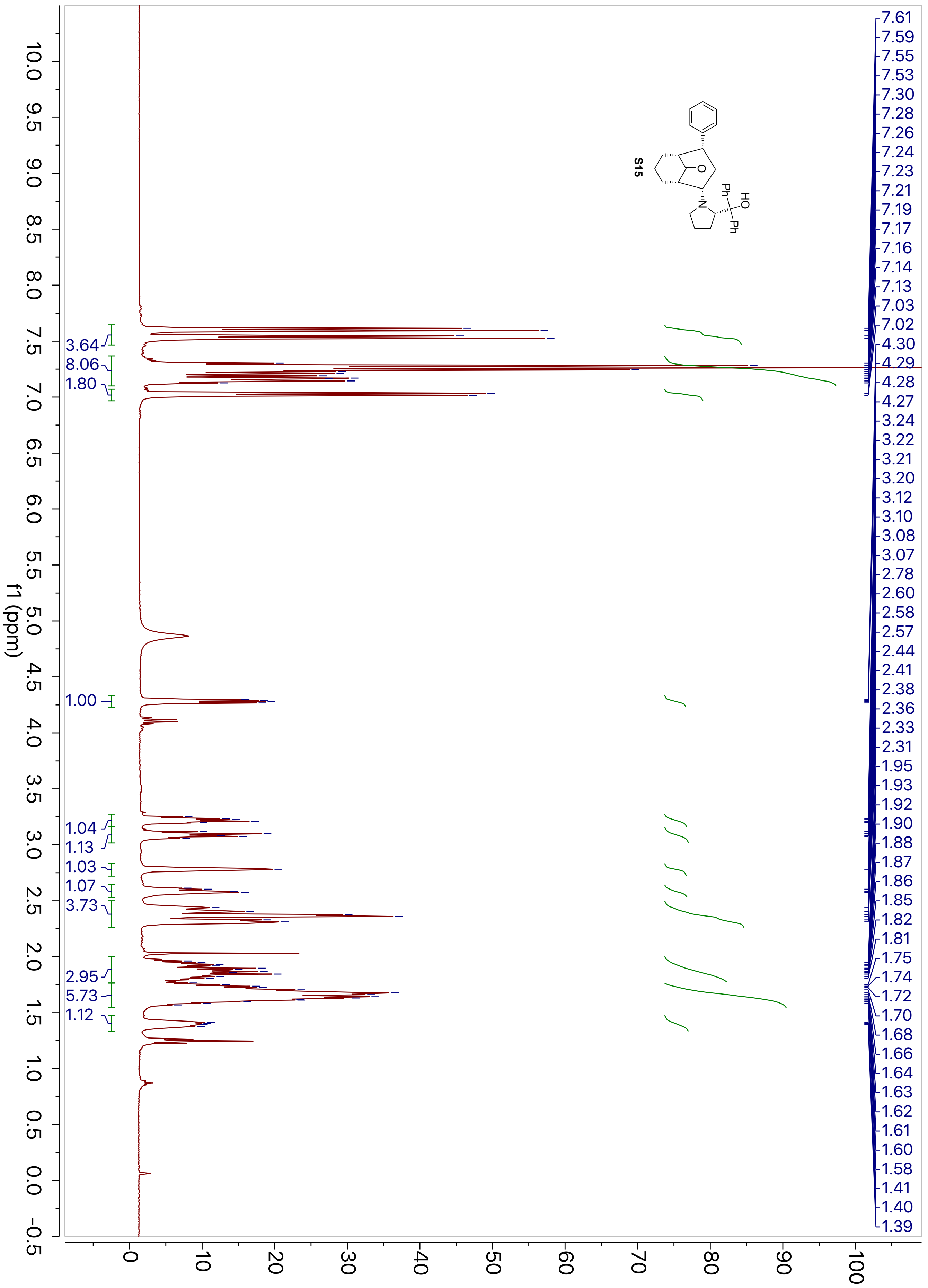
S15
HO
Ph
Ph
N
O
7.61
7.59
7.55
7.53
7.30
7.28
7.26
7.24
7.23
7.21
7.19
7.17
7.16
7.14
7.13
7.03
7.02
4.30
4.29
4.28
4.27
3.24
3.22
3.21
3.20
3.12
3.10
3.08
3.07
2.78
2.60
2.58
2.57
2.44
2.41
2.38
2.36
2.33
2.31
1.95
1.93
1.92
1.90
1.88
1.87
1.86
1.85
1.82
1.81
1.75
1.74
1.72
1.70
1.68
1.66
1.64
1.63
1.62
1.61
1.60
1.58
1.41
1.40
1.39
3.64
8.06
1.80
1.00
1.04
1.13
1.03
1.07
3.73
2.95
5.73
1.12
f1 (ppm)

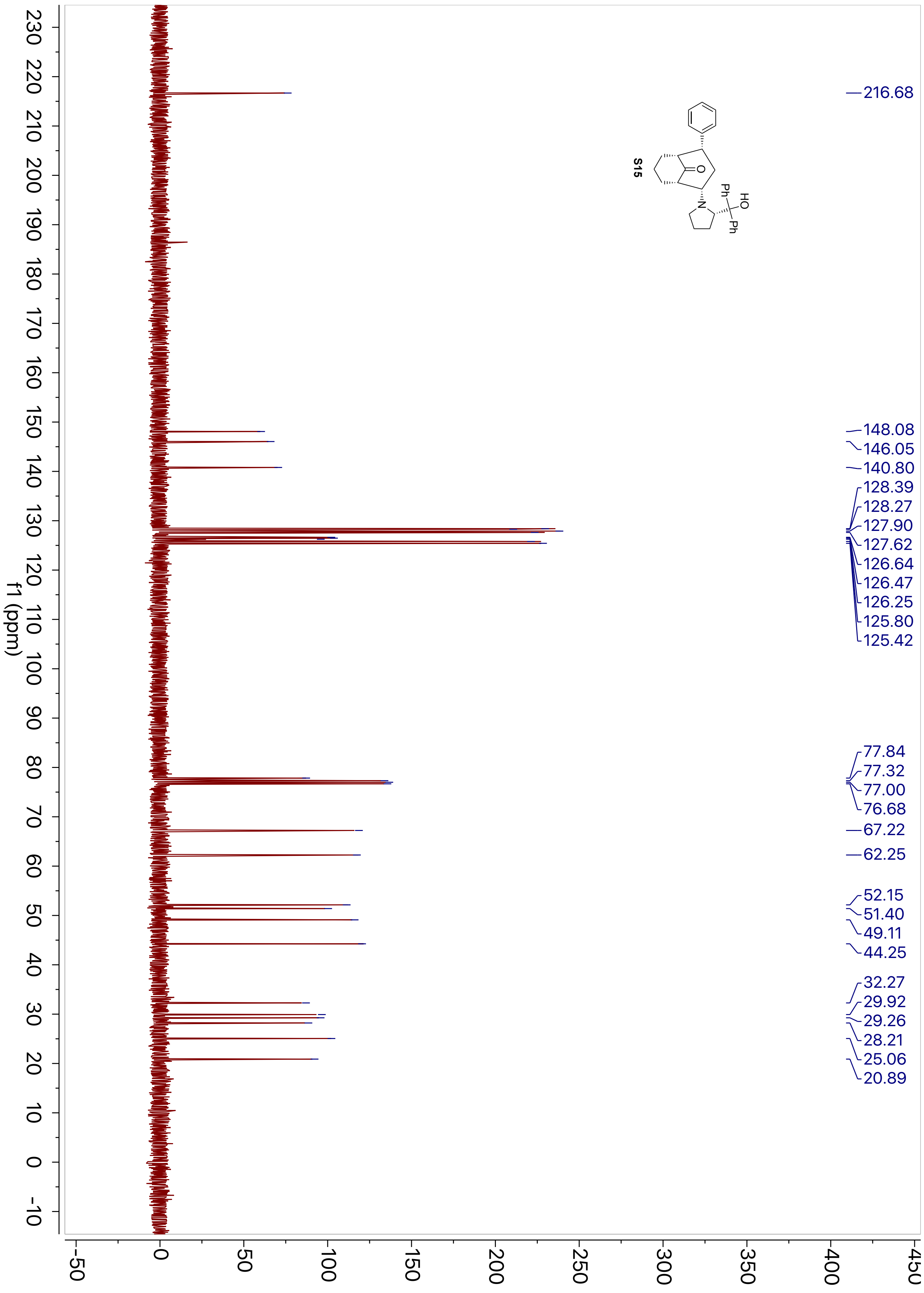
S15
HO
Ph
Ph
N
O
216.68
148.08
146.05
140.80
128.39
128.27
127.90
127.62
126.64
126.47
126.25
125.80
125.42
77.84
77.32
77.00
76.68
67.22
62.25
52.15
51.40
49.11
44.25
32.27
29.92
29.26
28.21
25.06
20.89
f1 (ppm)

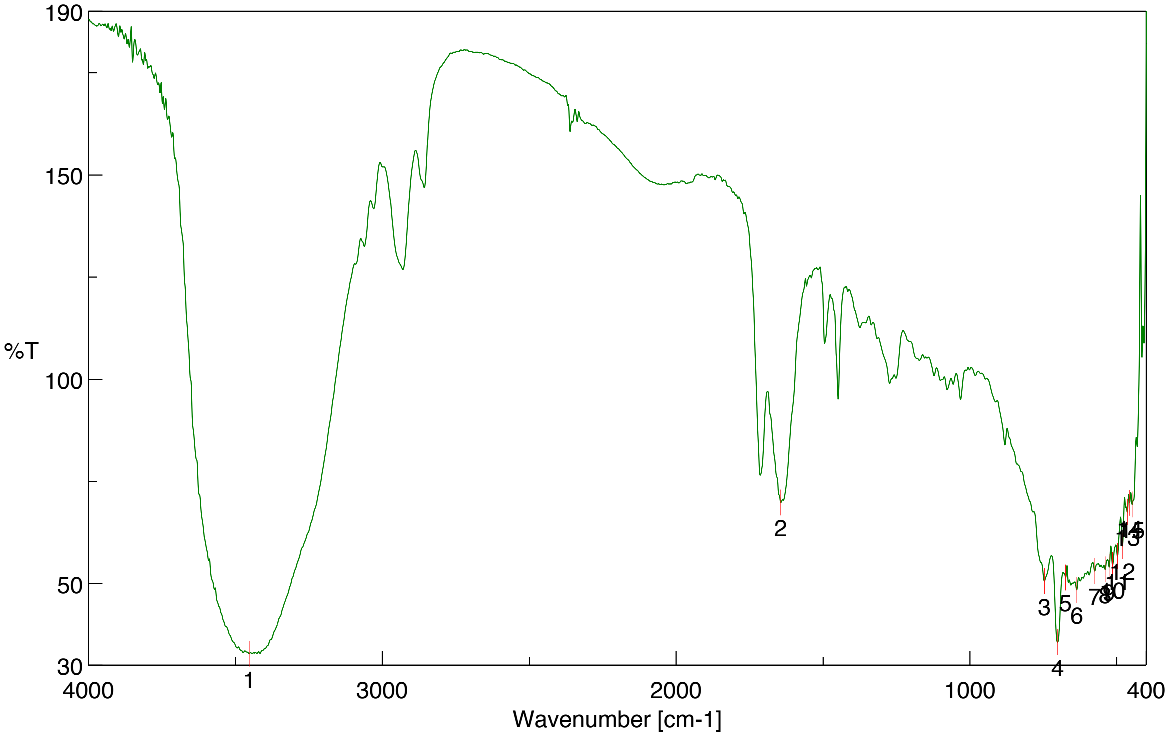

[ピーク検出結果] *

No.	位置 **	強度 ***	No.	位置 **	強度 ***	No.	位置 **	強度 ***
1	3452.92	32.8125	2	1644.02	69.8039	3	746.317	50.5921
4	700.998	35.6648	5	674.963	51.4851	6	636.394	48.4215
7	574.683	53.0504	8	539.971	53.4262	9	526.471	53.9844
10	514.901	54.5606	11	497.544	56.654	12	481.153	59.127
13	463.796	67.4505	14	456.082	69.7629	15	447.404	69.3148

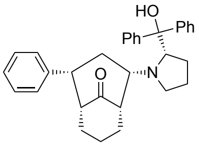

S15

* Detection result

** Wavenumber

*** Intensity

008109-10min#22-28 RT: 0.17-0.22 AV: 7
T: FTMS + p ESI Full ms [150.00-2000.00]
m/z= 242.0305-244.1380

m/z	Intensity	Relative
242.0792	183644.0	100.00
242.1077	6397.1	3.48
242.2848	32334.2	17.61
242.7632	17001.5	9.26
243.0830	6397.4	3.48
243.1066	64218.9	34.97
243.1621	36853.0	20.07
243.6019	9716.8	5.29
243.6057	9285.2	5.06
243.6470	5297.5	2.88
244.0831	39200.4	21.35

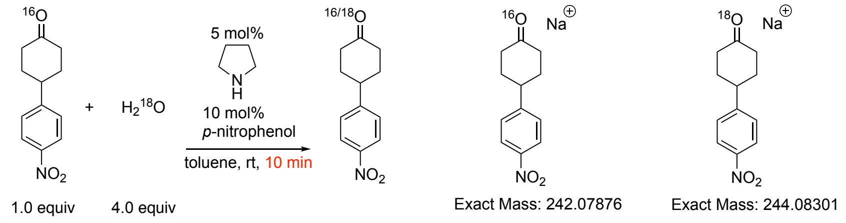

C:\Thermo\...\data\umekubo\008109-30min 2017/11/15 9:29:19

008109-30min#1-26 RT: 0.00-0.20 AV: 26
T: FTMS + p ESI Full ms [150.00-2000.00]
m/z= 242.0165-244.1357

m/z	Intensity	Relative
242.0787	4431526.5	89.35
242.1882	43861.2	0.88
242.2842	1176755.8	23.73
242.3115	51626.8	1.04
242.5696	48389.0	0.98
243.0689	55422.0	1.12
243.0820	208324.8	4.20
243.1080	237503.3	4.79
243.1621	45637.6	0.92
243.3036	44855.2	0.90
243.3479	45514.3	0.92
243.7246	42359.9	0.85
243.7808	45940.7	0.93
243.9676	39267.2	0.79
244.0829	4959886.5	100.00
244.1133	50992.7	1.03

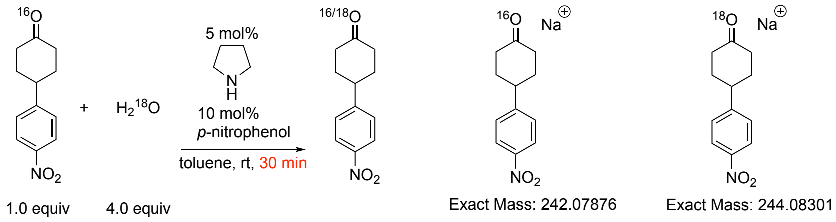

008109-45min#1-39 RT: 0.01-0.30 AV: 39
T: FTMS + p ESI Full ms [150.00-2000.00]
m/z= 242.0013-244.1022

m/z	Intensity	Relative
242.0788	737983.2	36.32
242.0895	26184.7	1.29
242.4145	35363.4	1.74
242.9701	33182.8	1.63
243.0139	28467.1	1.40
243.1071	98379.0	4.84
243.1611	35975.9	1.77
243.2868	31483.3	1.55
243.8755	22617.8	1.11
243.9828	26521.3	1.31
244.0830	2032041.6	100.00

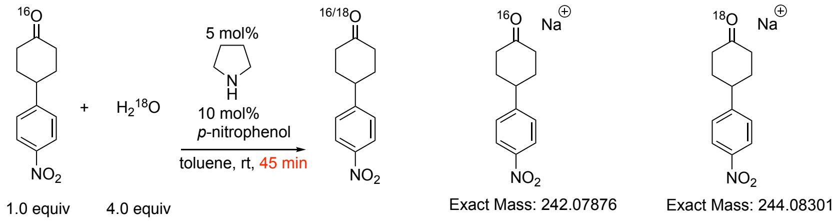

008109-60min#1-39 RT: 0.01-0.30 AV: 39
T: FTMS + p ESI Full ms [150.00-2000.00]
m/z= 242.0444-244.1726

m/z	Intensity	Relative
242.0788	2150500.5	41.38
242.2843	708039.9	13.63
242.2988	33839.0	0.65
242.9543	27625.2	0.53
243.1069	157037.8	3.02
243.1608	88893.4	1.71
243.6730	31046.1	0.60
244.0216	29816.3	0.57
244.0831	5196597.0	100.00
244.1087	24154.3	0.46

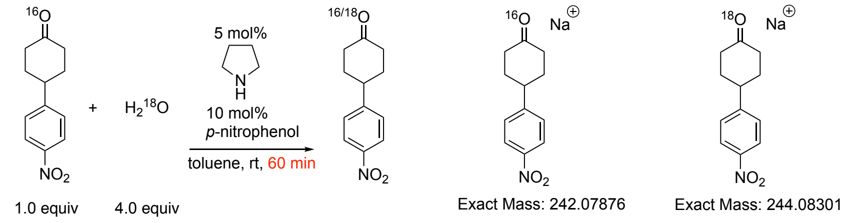

008109-90min#1-39 RT: 0.00-0.30 AV: 39
T: FTMS + p ESI Full ms [150.00-2000.00]
m/z= 242.0249-244.1554

m/z	Intensity	Relative
242.0285	29379.5	0.84
242.0790	1183342.9	33.89
242.2520	33758.7	0.97
242.2862	36732.7	1.05
243.0142	32225.2	0.92
243.0967	34327.1	0.98
243.1076	116538.9	3.34
243.1623	93557.0	2.68
243.4371	35419.1	1.01
243.8712	33566.5	0.96
243.8812	29816.8	0.85
243.9293	33199.1	0.95
244.0831	3492131.3	100.00
244.1283	33279.7	0.95
244.1390	28915.4	0.83

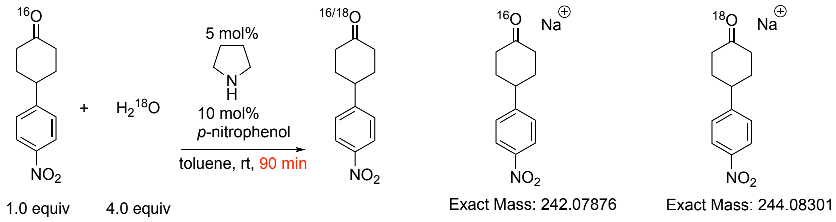

C:\Thermo\...\data\umekubo\008109-120min 2017/11/15 11:11:16

008109-120min#1-39 RT: 0.00-0.30 AV: 39
T: FTMS + p ESI Full ms [150.00-2000.00]
m/z= 242.0030-244.1560

m/z	Intensity	Relative
242.0285	29379.5	0.84
242.0790	1183342.9	33.89
242.2520	33758.7	0.97
242.2862	36732.7	1.05
243.0142	32225.2	0.92
243.0967	34327.1	0.98
243.1076	116538.9	3.34
243.1623	93557.0	2.68
243.4371	35419.1	1.01
243.8712	33566.5	0.96
243.8812	29816.8	0.85
243.9293	33199.1	0.95
244.0831	3492131.3	100.00
244.1283	33279.7	0.95
244.1390	28915.4	0.83

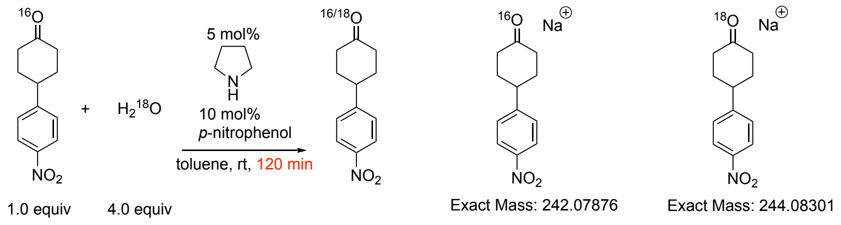

C:\Thermo\...\009042-1min_180304154252 2018/03/04 15:42:52

009042-1min_180304154252#18 RT: 0.14
T: FTMS + p ESI sid=25.00 Full ms [150.00-2000.00]
m/z= 243.4946-247.6496

m/z	Intensity	Relative
244.0940	79360.9	45.74
244.1045	173505.9	100.00
245.0962	30332.8	17.48
246.4555	23804.3	13.72
246.9221	38118.1	21.97

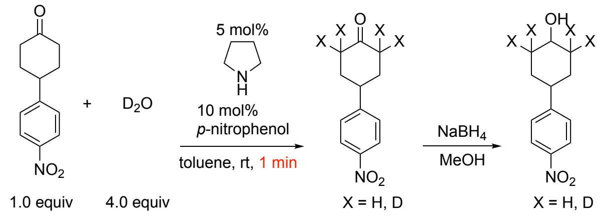

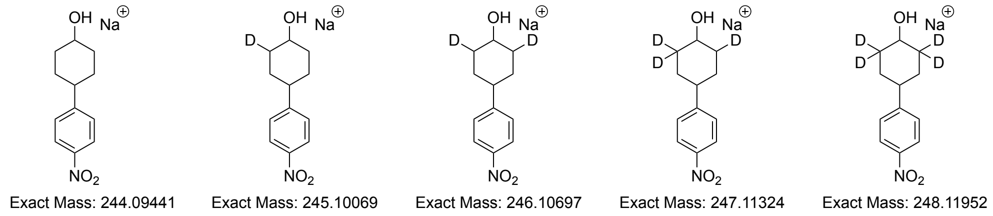

009042-3min_180304155107#1-38 RT: 0.00-0.30 AV: 38
T: FTMS + p ESI sid=25.00 Full ms [150.00-2000.00]
m/z= 243.4996-249.6443

m/z	Intensity	Relative
243.5004	909.4	0.55
244.0584	988.2	0.60
244.0941	164472.7	100.00
244.1044	157532.9	95.78
244.2284	780.4	0.47
245.0783	3793.0	2.31
245.1012	35330.5	21.48
245.3181	1378.2	0.84
245.4601	730.4	0.44
246.1069	21004.1	12.77
246.9226	7426.3	4.52
246.9707	838.4	0.51
247.0695	1503.0	0.91
247.0928	746.8	0.45
247.1130	2319.6	1.41
247.8159	777.6	0.47
248.9197	1899.8	1.16
249.1229	13078.0	7.95
249.1453	1986.1	1.21
249.1818	1300.3	0.79

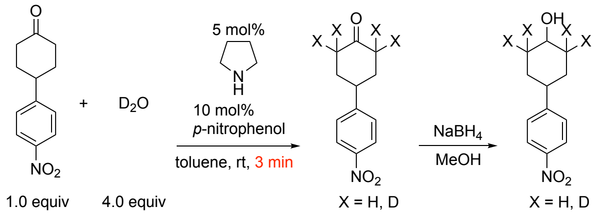

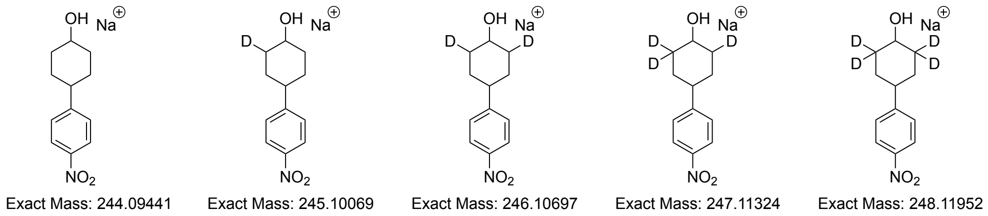

009042-5min#1-37 RT: 0.00-0.30 AV: 37
T: FTMS + p ESI sid=25.00 Full ms [150.00-2000.00]
m/z= 243.5377-249.7054

m/z	Intensity	Relative
243.9733	432.9	0.41
244.0943	86414.8	82.49
244.1045	104762.4	100.00
244.7808	427.2	0.41
245.0781	7664.4	7.32
245.1012	45857.7	43.77
245.5139	430.7	0.41
246.1070	45448.0	43.38
246.9224	6990.3	6.67
246.9329	1344.7	1.28
247.0315	509.1	0.49
247.0573	1047.3	1.00
247.0707	615.7	0.59
247.1131	19580.9	18.69
248.9196	1836.7	1.75
249.1096	452.7	0.43
249.1230	9872.9	9.42
249.1455	3021.9	2.88
249.1821	10419.8	9.95
249.2058	1706.1	1.63

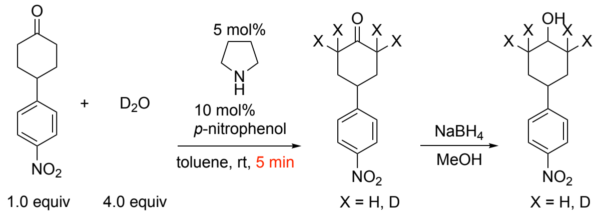

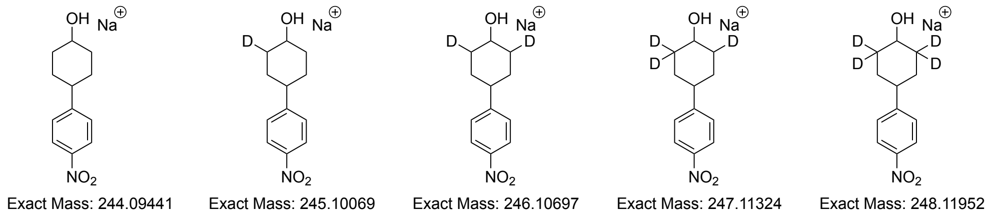

009042-10min#1-38 RT: 0.00-0.30 AV: 38
T: FTMS + p ESI sid=25.00 Full ms [150.00-2000.00]
m/z= 243.6055-248.2561

m/z	Intensity	Relative
244.0826	544.2	0.26
244.0942	26639.0	12.50
244.1044	213176.0	100.00
244.1269	2222.6	1.04
244.9709	526.0	0.25
244.9815	622.3	0.29
245.0776	1137.4	0.53
245.1007	21112.3	9.90
245.1983	676.1	0.32
246.1067	40947.0	19.21
246.1328	1623.3	0.76
246.9225	2925.8	1.37
246.9331	2599.3	1.22
247.0568	563.6	0.26
247.1130	24268.3	11.38
247.1222	927.8	0.44
247.1432	1553.4	0.73
247.3512	564.0	0.26
247.7398	557.5	0.26
248.1197	1213.1	0.57

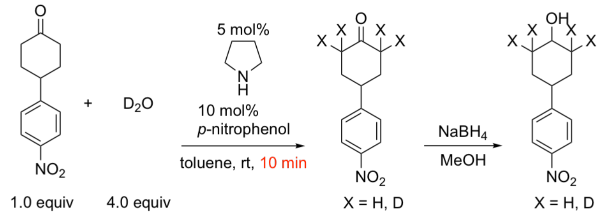

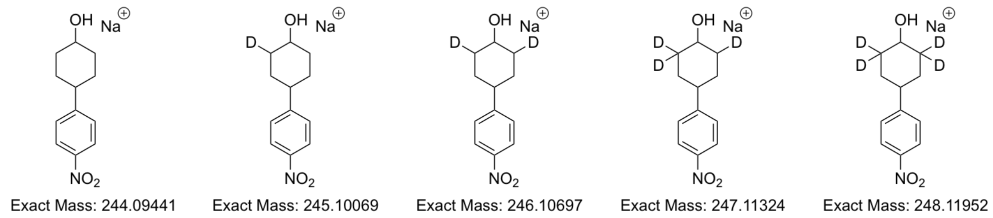

009042-15min#19 RT: 0.15
T: FTMS + p ESI sid=25.00 Full ms [150.00-2000.00]
m/z= 243.7036-248.3289

m/z	Intensity	Relative
244.0941	17927.9	12.56
244.1043	142792.4	100.00
245.0964	13178.5	9.23
245.1012	29235.5	20.47
246.1066	33536.6	23.49
247.1129	44035.6	30.84

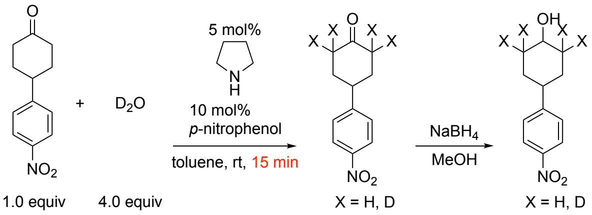

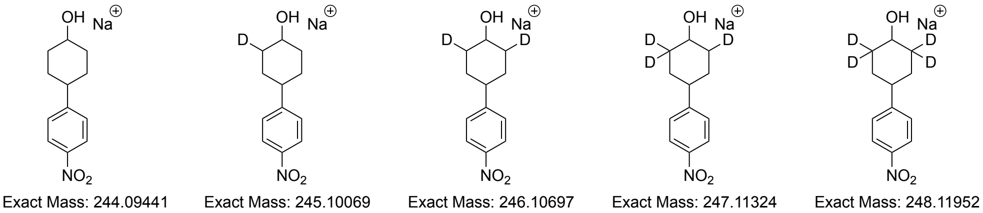

009042-20min#7 RT: 0.05
T: FTMS + p ESI sid=25.00 Full ms [150.00-2000.00]
m/z= 243.7245-248.3861

m/z	Intensity	Relative
244.0943	62193.9	23.04
244.1044	269903.8	100.00
244.1159	39770.8	14.74
245.0998	53684.5	19.89
246.1065	54357.9	20.14
247.1132	76399.5	28.31
247.2128	85825.2	31.80

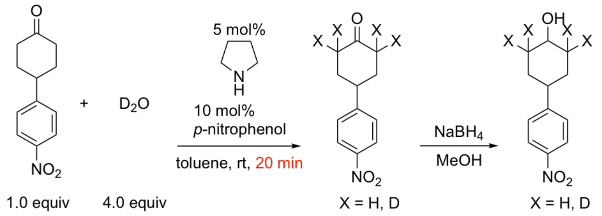

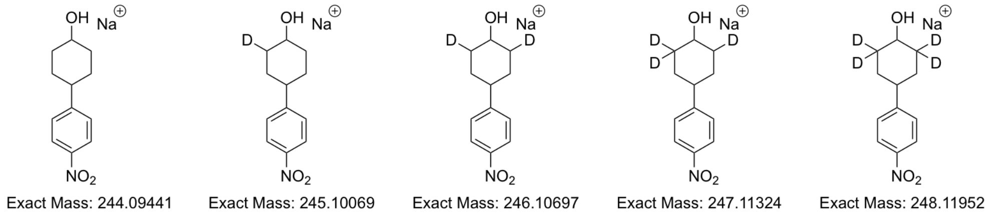

009042-30min#1-37 RT: 0.01-0.29 AV: 37
T: FTMS + p ESI sid=25.00 Full ms [150.00-2000.00]
m/z= 243.8602-247.5363

m/z	Intensity	Relative
244.0587	654.4	0.30
244.0946	5207.2	2.36
244.1043	220344.2	100.00
244.1502	720.0	0.33
244.7518	599.2	0.27
244.8202	658.0	0.30
244.9858	683.7	0.31
245.0778	4581.4	2.08
245.1007	12205.9	5.54
245.3354	711.8	0.32
245.6229	664.1	0.30
245.6363	680.4	0.31
246.1066	54968.3	24.95
246.1323	792.2	0.36
246.1683	1406.8	0.64
246.4820	652.7	0.30
246.9222	2028.2	0.92
247.1130	70764.8	32.12
247.1650	776.3	0.35
247.2408	762.6	0.35

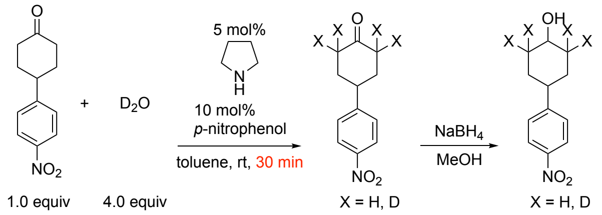

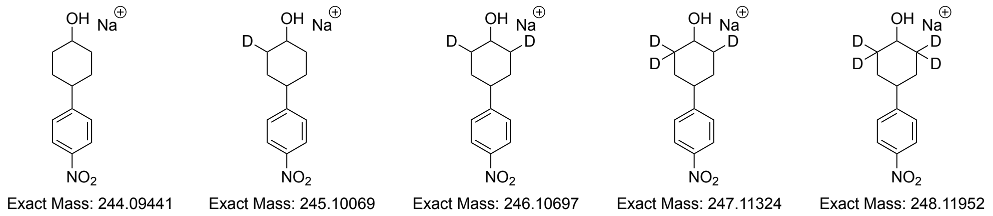

NuUm-Enolate-1min#1 RT: 0.01
T: FTMS + p ESI Full ms [150.00-2000.00]
m/z= 242.6587-254.6210

m/z	Intensity	Relative
244.0949	423312.3	100.00
245.0979	78955.7	18.65
254.2488	129513.0	30.60

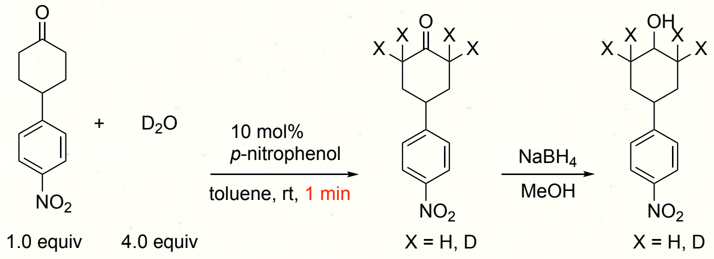

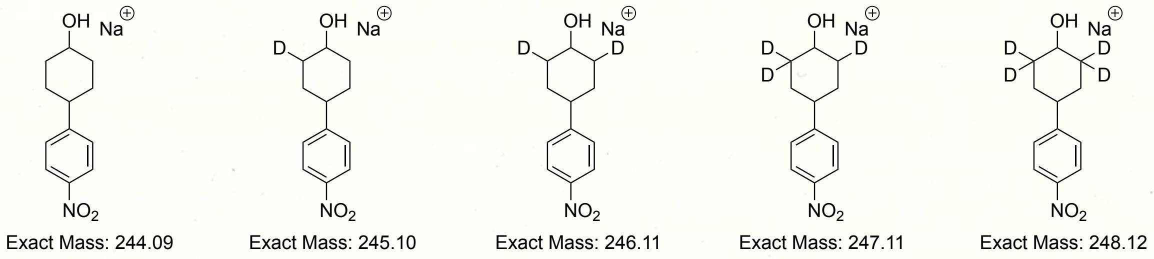

C:\Thermo\...\umekubo\NuUm-Enolate-3min 2020/04/12 9:58:42

NuUm-Enolate-3min#1 RT: 0.00
T: FTMS + p ESI Full ms [150.00-2000.00]
m/z= 242.9727-254.2210

m/z	Intensity	Relative
244.0944	348406.7	100.00
244.8869	18335.9	5.26
245.0979	48499.1	13.92
253.8098	20369.7	5.85
253.9509	24575.8	7.05

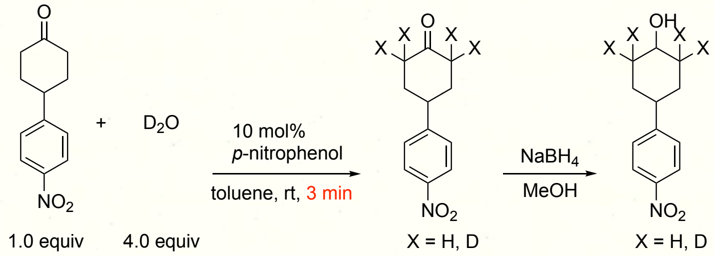

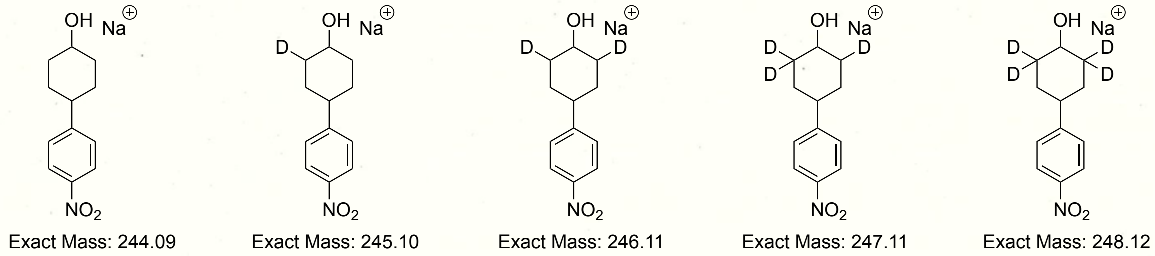

NuUm-Enolate-5min#1 RT: 0.00
T: FTMS + p ESI Full ms [150.00-2000.00]
m/z= 243.8251-253.2101

m/z	Intensity	Relative
244.0943	1089989.9	100.00
245.0974	113449.2	10.41
247.1440	67322.0	6.18
247.6354	72335.5	6.64
252.9002	74270.6	6.81

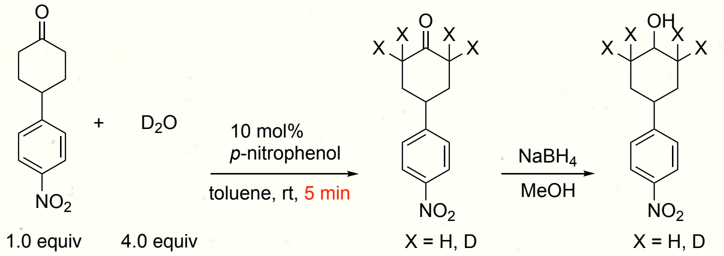

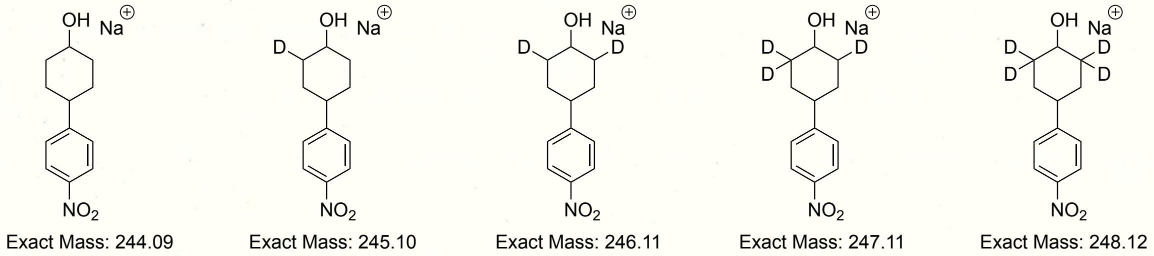

C:\Thermo\...\umekubo\NuUm-Enolate-10min 2020/04/12 10:08:59

NuUm-Enolate-10min#1 RT: 0.01
T: FTMS + p ESI Full ms [150.00-2000.00]
m/z= 243.2636-252.3160

m/z	Intensity	Relative
244.0943	1086124.0	100.00
245.0980	137894.1	12.70

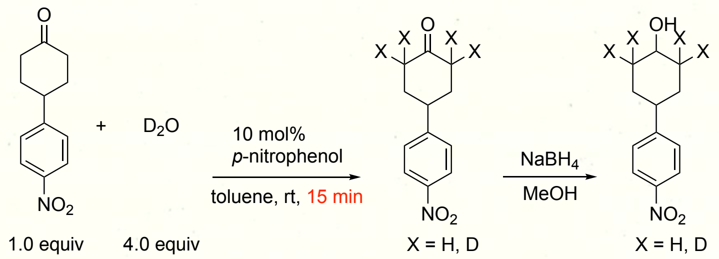

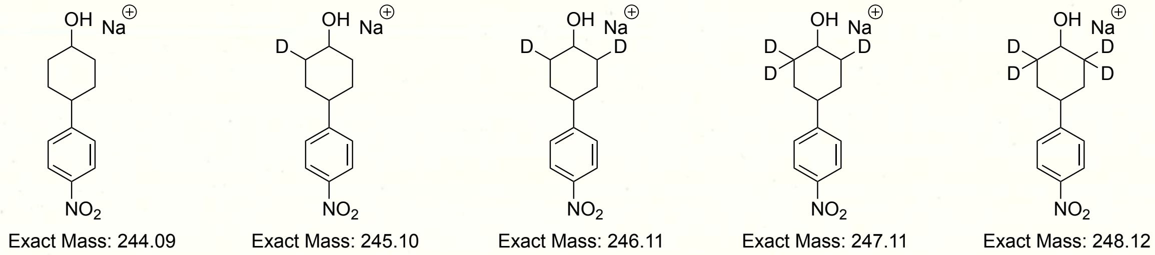

C:\Thermo\...\umekubo\NuUm-Enolate-20min 2020/04/12 11:40:35

NuUm-Enolate-20min#1 RT: 0.01
T: FTMS + p ESI Full ms [150.00-2000.00]
m/z= 243.7406-250.6357

m/z	Intensity	Relative
244.0944	202725.8	100.00
245.0791	26365.6	13.01
247.1790	18992.9	9.37
249.2934	23385.2	11.54
250.4592	19826.7	9.78

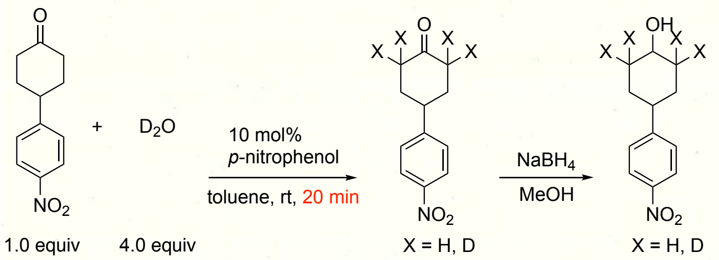

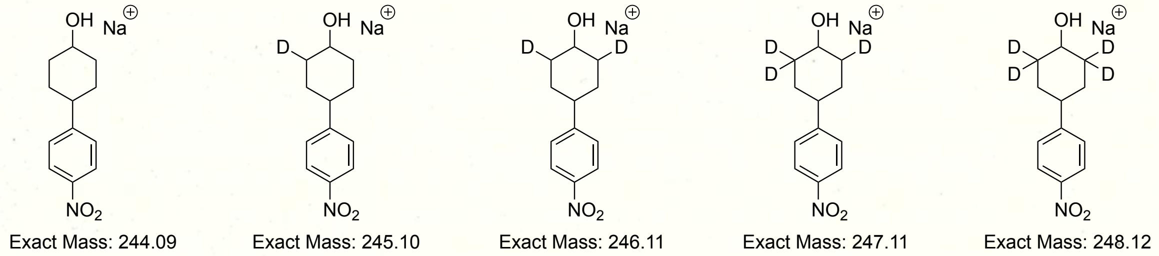

C:\Thermo\...\umekubo\NuUm-Enolate-30min   2020/04/12 11:40:46

NuUm-Enolate-30min#1 RT: 0.00
T: FTMS + p ESI Full ms [150.00-2000.00]
m/z= 242.9877-254.6004

m/z	Intensity	Relative
244.0948	1831223.1	100.00
253.0932	101269.8	5.53
253.9343	153473.8	8.38

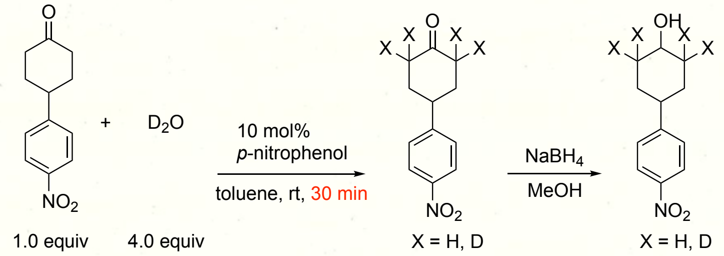

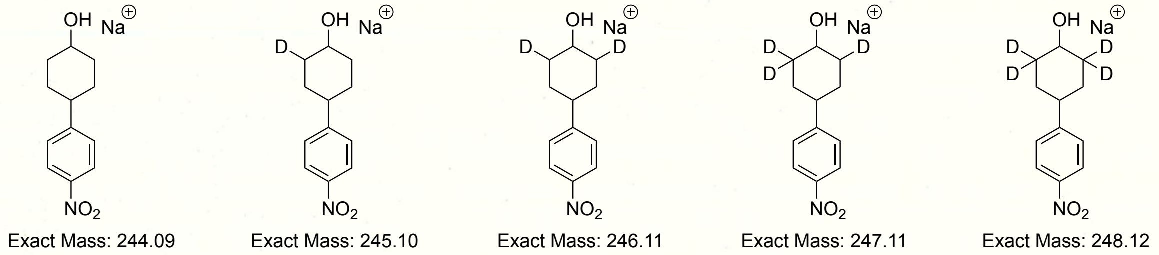

C:\Thermo\...\NuUm-Enolate-2-3min 2020/04/12 11:41:16

NuUm-Enolate-2-3min#1 RT: 0.00
T: FTMS + p ESI Full ms [150.00-2000.00]
m/z= 243.6754-250.5785

m/z	Intensity	Relative
244.0945	724302.4	100.00
250.1947	80913.8	11.17

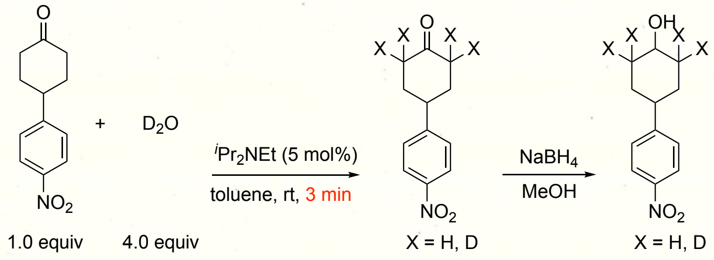

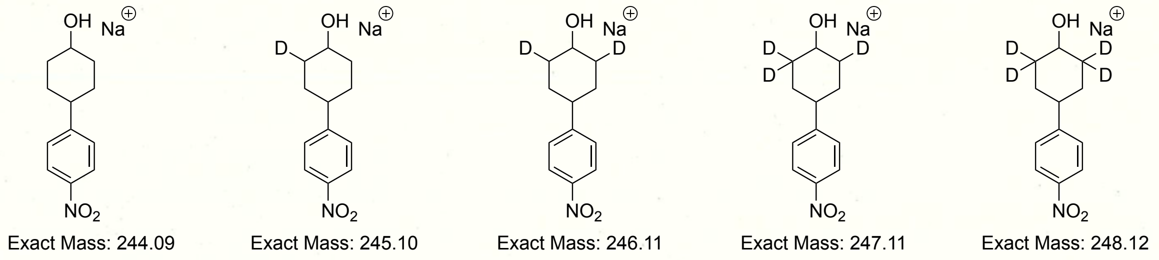

NuUm-Enolate-2-5min#1 RT: 0.01
T: FTMS + p ESI Full ms [150.00-2000.00]
m/z= 243.9446-253.4254

m/z	Intensity	Relative
244.0945	941727.8	100.00
246.4041	81673.7	8.67
253.1191	102182.4	10.85

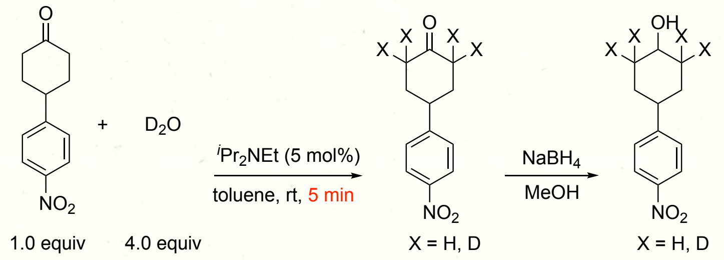

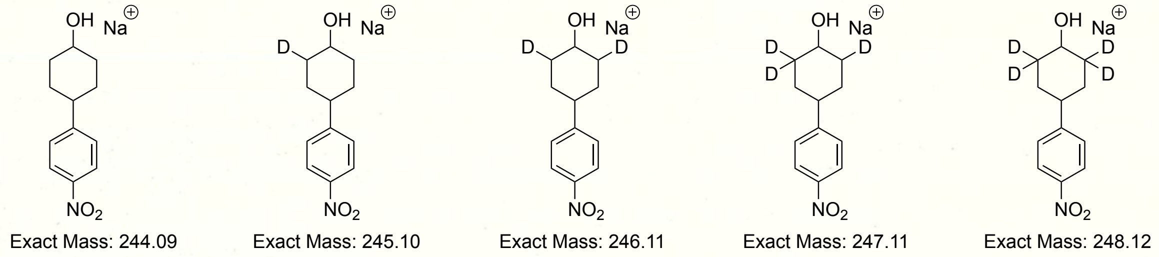

NuUm-Enolate-2-10min_200412114138 2020/04/12 11:41:53

NuUm-Enolate-2-10min_200412114138#1 RT: 0.01
T: FTMS + p ESI Full ms [150.00-2000.00]
m/z= 242.6142-267.7911

m/z	Intensity	Relative
244.0947	704731.2	100.00
247.6630	91645.7	13.00
267.2563	147938.7	20.99

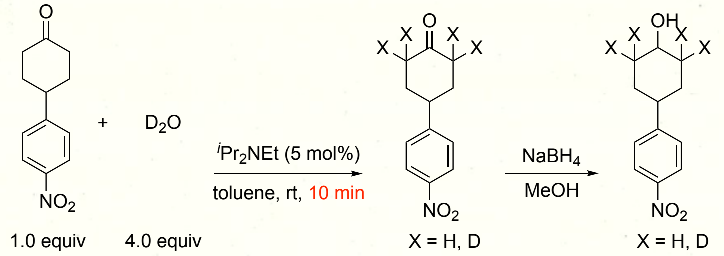

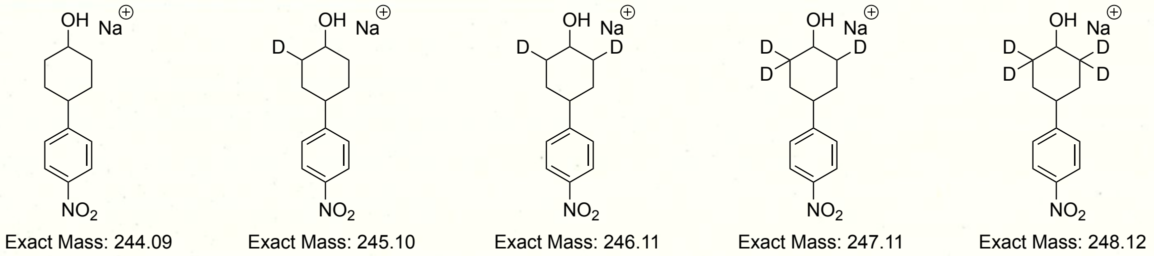

NuUm-Enolate-2-15min_200412114138 2020/04/12 11:42:13

NuUm-Enolate-2-15min_200412114138#1 RT: 0.01
T: FTMS + p ESI Full ms [150.00-2000.00]
m/z= 243.5469-253.5809

m/z	Intensity	Relative
244.0946	815458.6	100.00
245.9832	83565.6	10.25
249.0116	91196.5	11.18
253.1181	111539.8	13.68

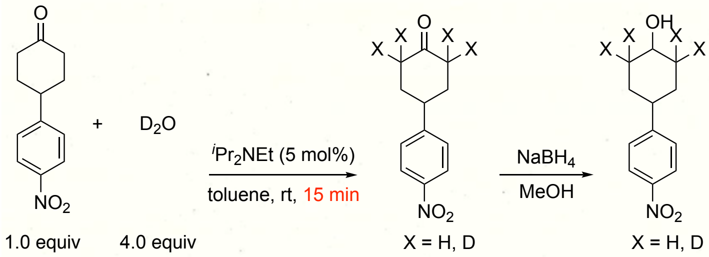

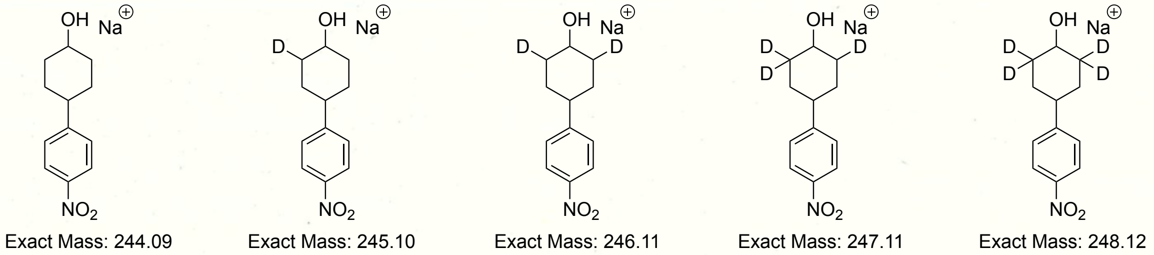

NuUm-Enolate-2-20min_200412114138 2020/04/12 11:42:38

NuUm-Enolate-2-20min_200412114138#1 RT: 0.01
T: FTMS + p ESI Full ms [150.00-2000.00]
m/z= 243.3689-252.8813

m/z	Intensity	Relative
244.0946	689974.7	100.00
251.6744	86658.0	12.56

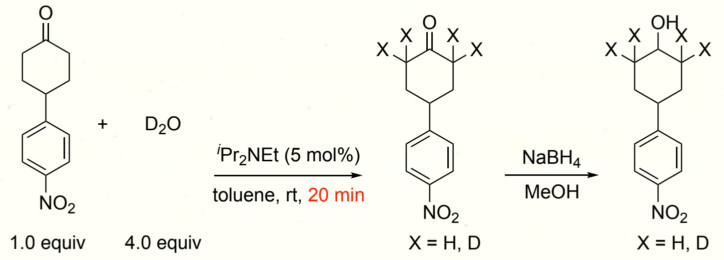

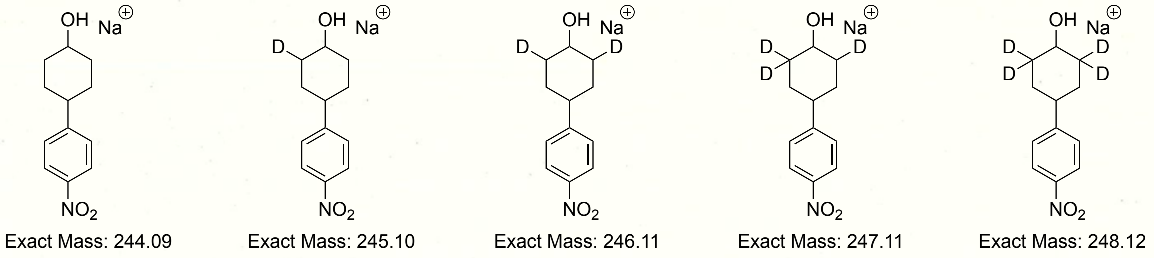

NuUm-Enolate-2-30min_200412114138#1 RT: 0.01
T: FTMS + p ESI Full ms [150.00-2000.00]
m/z= 243.3172-253.4315

m/z	Intensity	Relative
244.0946	589362.2	100.00
253.1175	92765.4	15.74

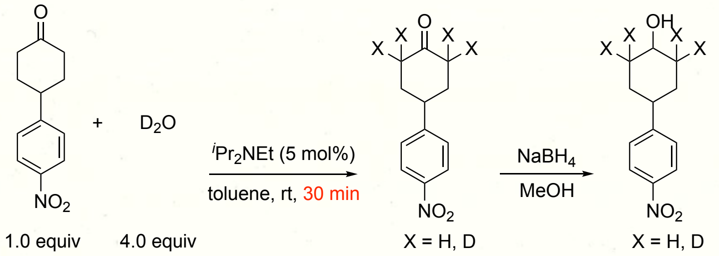

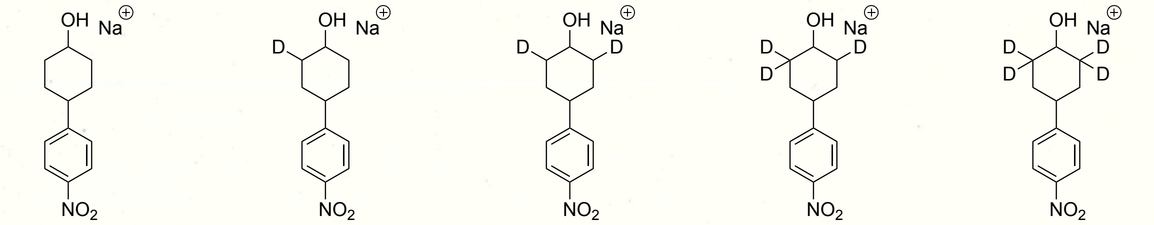

Exact Mass: 244.09 Exact Mass: 245.10 Exact Mass: 246.11 Exact Mass: 247.11 Exact Mass: 248.12

NuUm-Enolate-3-1min_200412114138 2020/04/12 13:58:35

NuUm-Enolate-3-1min_200412114138#2-128 RT: 0.01-1.01 AV: 127
T: FTMS + p ESI Full ms [150.00-2000.00]
m/z= 243.4596-249.9547

m/z	Intensity	Relative
244.0945	212808.6	76.61
244.2120	881.6	0.32
244.5761	1480.0	0.53
244.8481	1027.7	0.37
244.9760	1185.6	0.43
245.1014	21653.8	7.79
245.1996	1448.3	0.52
245.3847	1023.1	0.37
245.4767	886.1	0.32
246.1071	24514.4	8.82
246.2077	61500.0	22.14
247.1061	921.1	0.33
247.1137	5855.2	2.11
247.2140	214782.7	77.32
247.4841	1148.3	0.41
248.1198	1913.3	0.69
248.2203	277798.6	100.00
248.4317	877.5	0.32
249.2269	106025.1	38.17
249.8418	1078.8	0.39

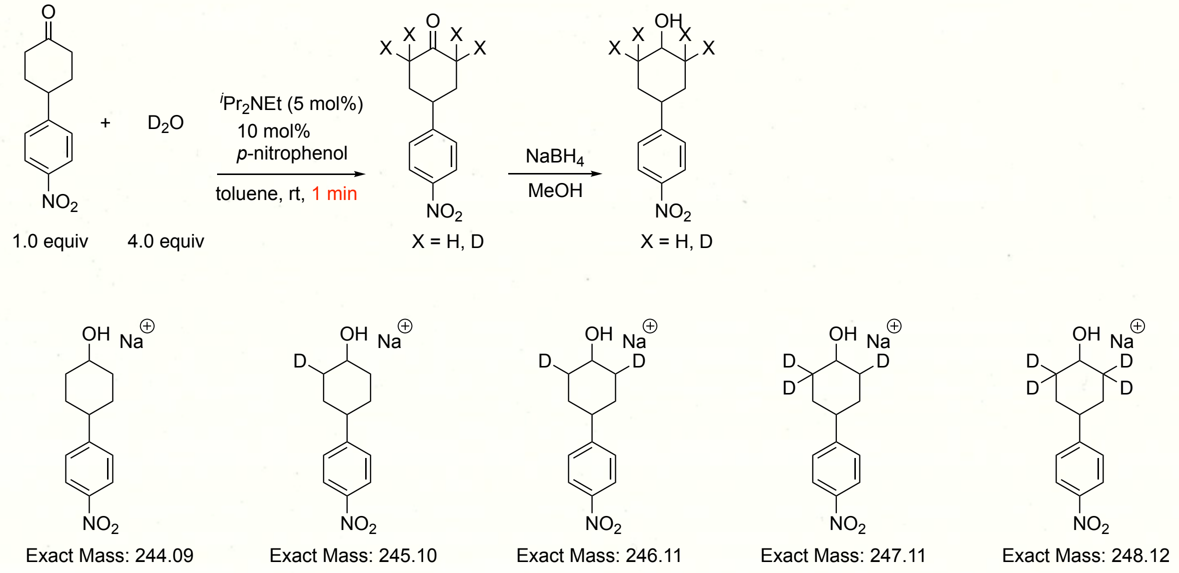

C:\Thermo\...\NuUm-Enolate-3-10min 2020/04/12 14:14:49

NuUm-Enolate-3-10min#1-130 RT: 0.00-1.01 AV: 130
T: FTMS + p ESI Full ms [150.00-2000.00]
m/z= 243.7251-249.6445

m/z	Intensity	Relative
243.8165	2240.6	0.13
243.8851	1451.8	0.09
243.9551	2464.5	0.15
243.9670	1465.1	0.09
244.0945	25312.5	1.51
244.4219	1555.7	0.09
245.1021	5084.3	0.30
245.2388	1521.3	0.09
245.3932	2373.9	0.14
246.1071	123902.9	7.38
246.2078	72276.4	4.31
246.4701	2432.3	0.14
247.1135	239738.0	14.29
247.2140	708872.4	42.24
247.9235	2079.9	0.12
248.1199	94397.4	5.63
248.2203	1678146.9	100.00
248.4613	2175.7	0.13
249.1007	1486.8	0.09
249.2268	1508096.0	89.87

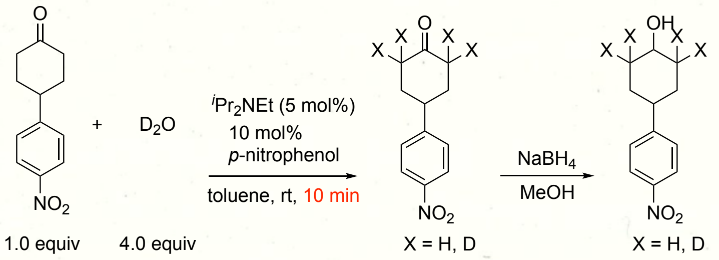

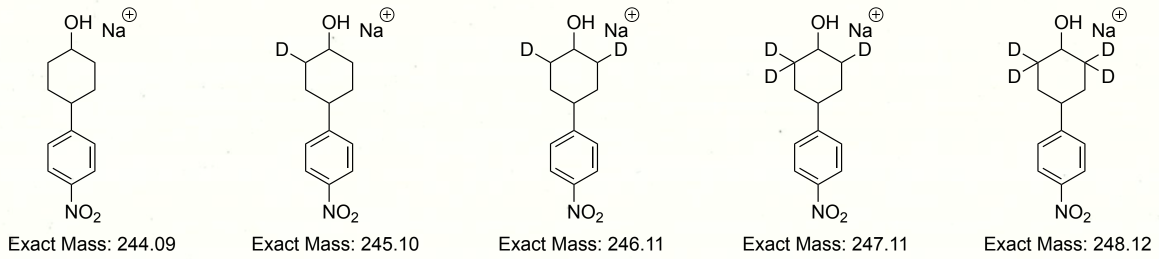

C:\Thermo\...\NuUm-Enolate-3-30min 2020/04/12 15:46:36

NuUm-Enolate-3-30min#2-256 RT: 0.01-2.00 AV: 255
T: FTMS + p ESI Full ms [150.00-2000.00]
m/z= 241.2370-250.5830

m/z	Intensity	Relative
242.1579	3493.2	0.06
242.2843	1761.4	0.03
243.0612	1271.3	0.02
243.5558	1394.5	0.02
244.1246	1676.5	0.03
245.1602	1378.9	0.02
245.1986	1489.1	0.02
246.1073	3733.1	0.06
246.1127	1305.9	0.02
246.2077	302791.4	4.94
246.3125	1241.7	0.02
247.1131	22675.7	0.37
247.2139	2236366.8	36.48
247.7315	1300.6	0.02
248.1197	8377.8	0.14
248.2201	6129802.0	100.00
249.1114	1309.4	0.02
249.2067	1575.1	0.03
249.2265	5922013.5	96.61
250.2295	819884.4	13.38

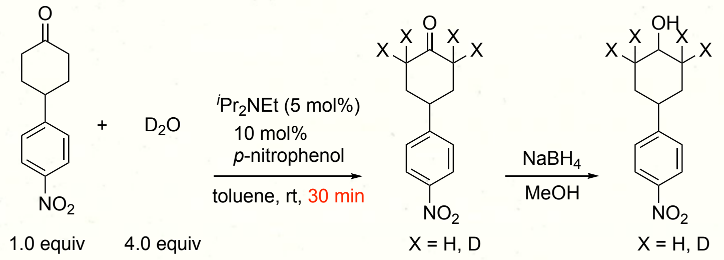

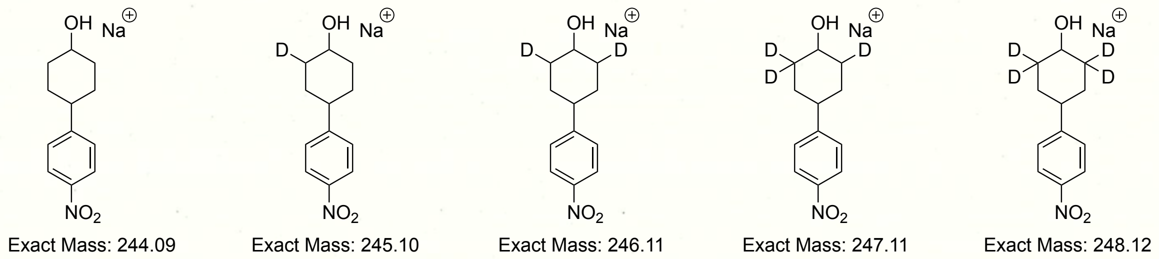

X-ray Structure Report

for

01

May 18, 2018

Experimental

Data Collection

A colorless prism crystal of $C_{32}H_{35}NO_2$ having approximate dimensions of 0.400 x 0.150 x 0.150 mm was mounted on a glass fiber. All measurements were made on a Rigaku XtaLAB mini diffractometer using graphite monochromated Mo-Kα radiation.

The crystal-to-detector distance was 50.00 mm.

Cell constants and an orientation matrix for data collection corresponded to a primitive monoclinic cell with dimensions:

a	=	10.3803(15) Å			
b	=	6.7239(10) Å	β	=	97.454(7)^{o}
c	=	18.224(3) Å			
V	=	1261.2(3) Å^{3}			

For Z = 2 and F.W. = 465.63, the calculated density is 1.226 g/cm^{3}. Based on the reflection conditions of:

0k0: k = 2n

packing considerations, a statistical analysis of intensity distribution, and the successful solution and refinement of the structure, the space group was determined to be:

$P2_1$ (#4)

The data were collected at a temperature of -123 $\pm$ 1°C to a maximum 2θ value of 54.9^{o}. A total of 540 oscillation images were collected. A sweep of data was done using ω scans from -60.0 to 120.0^{o} in 1.00^{o} step, at χ=54.0^{o} and ϕ = 0.0^{o}. The exposure rate was 24.0 [sec./^{o}]. The detector swing angle was 30.00^{o}. A second sweep was performed using ω scans from -60.0 to 120.0^{o} in 1.00^{o} step, at χ=54.0^{o} and ϕ = 120.0^{o}. The exposure rate was 24.0 [sec./^{o}]. The detector swing angle was 30.00^{o}. Another sweep was performed using ω scans from -60.0 to 120.0^{o} in 1.00^{o} step, at χ=54.0^{o} and ϕ = 240.0^{o}. The exposure rate was 24.0 [sec./^{o}]. The detector swing angle

was 30.00^o. Another sweep was performed using ω scans from -60.0 to 120.0^o in 1.00^o step, at χ=54.0^o and ϕ = 0.0^o. The exposure rate was 24.0 [sec./^{o}]. The detector swing angle was 30.00^o. Another sweep was performed using ω scans from -60.0 to 120.0^o in 1.00^o step, at χ=54.0^o and ϕ = 120.0^o. The exposure rate was 24.0 [sec./^{o}]. The detector swing angle was 30.00^o. Another sweep was performed using ω scans from -60.0 to 120.0^o in 1.00^o step, at χ=54.0^o and ϕ = 240.0^o. The exposure rate was 24.0 [sec./^{o}]. The detector swing angle was 30.00^o. The crystal-to-detector distance was 50.00 mm. Readout was performed in the 0.073 mm pixel mode.

Data Reduction

Of the 13313 reflections were collected, where 5746 were unique (R_{int} = 0.0466). Data were collected and processed using CrystalClear (Rigaku). [1]

The linear absorption coefficient, μ, for Mo-Kα radiation is 0.751 cm^{-1}. An empirical absorption correction was applied which resulted in transmission factors ranging from 0.823 to 0.989. The data were corrected for Lorentz and polarization effects.

Structure Solution and Refinement

The structure was solved by direct methods[2] and expanded using Fourier techniques. The non-hydrogen atoms were refined anisotropically. Hydrogen atoms were refined using the riding model. The final cycle of full-matrix least-squares refinement[3] on F^2 was based on 5746 observed reflections and 316 variable parameters and converged (largest parameter shift was 0.00 times its esd) with unweighted and weighted agreement factors of:

$$R1 = \Sigma \,||Fo| - |Fc|| \,/\, \Sigma\, |Fo| = 0.0450$$

$$wR2 = [\, \Sigma \,(\, w\, (Fo^2 - Fc^2)^2\,)/ \,\Sigma\, w(Fo^2)^2]^{1/2} = 0.1003$$

The goodness of fit[4] was 1.02. Unit weights were used. The maximum and minimum peaks on the final difference Fourier map corresponded to 0.20 and -0.24 $e^-/Å^3$, respectively. The final Flack parameter [5] was -0.6(8), indicating that inversion-distinguishing power is too weak.[6] It is required to avarage Friedel pairs and do least-squares structure refinement again.

Neutral atom scattering factors were taken from International Tables for

Crystallography (IT), Vol. C, Table 6.1.1.4 [7]. Anomalous dispersion effects were included in Fcalc[8]; the values for Δf' and Δf" were those of Creagh and McAuley[9]. The values for the mass attenuation coefficients are those of Creagh and Hubbell[10]. All calculations were performed using the CrystalStructure[11] crystallographic software package except for refinement, which was performed using SHELXL Version 2013/4[12].

References

(1) CrystalClear: Data Collection and Processing Software, Rigaku Corporation (1998-2015). Tokyo 196-8666, Japan.

(2) SIR2014: Burla, M. C., Caliandro, R., Carrozzini, B., Cascarano, G. L., Giacovazzo, C., Mallamo, M., Mazzone, A., Polidori, G. (2014). In preparation.

(3) Least Squares function minimized: (SHELXL Version 2013/4)

$\Sigma w(F_o^2-F_c^2)^2$ where w = Least Squares weights.

(4) Goodness of fit is defined as:

$[\Sigma w(F_o^2-F_c^2)^2/(N_o-N_v)]^{1/2}$

where: N_o = number of observations
N_v = number of variables

(5) Parsons, S. and Flack, H. (2004), Acta Cryst. A60, s61.

(6) Flack, H.D. and Bernardinelli (2000), J. Appl. Cryst. 33, 114-1148.

(7) International Tables for Crystallography, Vol.C (1992). Ed. A.J.C. Wilson, Kluwer Academic Publishers, Dordrecht, Netherlands, Table 6.1.1.4, pp. 572.

(8) Ibers, J. A. & Hamilton, W. C.; Acta Crystallogr., 17, 781 (1964).

(9) Creagh, D. C. & McAuley, W.J .; "International Tables for Crystallography", Vol C, (A.J.C. Wilson, ed.), Kluwer Academic Publishers, Boston, Table 4.2.6.8, pages 219-222 (1992).

(10) Creagh, D. C. & Hubbell, J.H..; "International Tables for Crystallography", Vol C, (A.J.C. Wilson, ed.), Kluwer Academic Publishers, Boston, Table 4.2.4.3, pages

200-206 (1992).

(11) CrystalStructure 4.3: Crystal Structure Analysis Package, Rigaku Corporation (2000-2018). Tokyo 196-8666, Japan.

(12) SHELXL Version 2013/4: Sheldrick, G. M. (2008). Acta Cryst. A64, 112-122.

EXPERIMENTAL DETAILS

A. Crystal Data

Empirical Formula	$C_{32}H_{35}NO_2$
Formula Weight	465.63
Crystal Color, Habit	colorless, prism
Crystal Dimensions	0.400 X 0.150 X 0.150 mm
Crystal System	monoclinic
Lattice Type	Primitive
Lattice Parameters	a = 10.3803(15) Å<br>b = 6.7239(10) Å<br>c = 18.224(3) Å<br>β = 97.454(7) $^{\circ}$<br>V = 1261.2(3) $Å^3$
Space Group	$P2_1$ (#4)
Z value	2
D_{calc}	1.226 g/cm^3
F_{000}	500.00
$\mu(MoK\alpha)$	0.751 cm^{-1}

B. Intensity Measurements

Diffractometer	XtaLAB mini
Radiation	MoKα (λ = 0.71075 Å)<br>graphite monochromated
Voltage, Current	50kV, 12mA
Temperature	-123.0^{o}C
Detector Aperture	75.0 mm (diameter)
Data Images	540 exposures
ω oscillation Range (χ=54.0, ϕ=0.0)	-60.0 - 120.0^{o}
Exposure Rate	24.0 sec./^{o}
Detector Swing Angle	30.00^{o}
ω oscillation Range (χ=54.0, ϕ=120.0)	-60.0 - 120.0^{o}
Exposure Rate	24.0 sec./^{o}
Detector Swing Angle	30.00^{o}
ω oscillation Range (χ=54.0, ϕ=240.0)	-60.0 - 120.0^{o}
Exposure Rate	24.0 sec./^{o}
Detector Swing Angle	30.00^{o}
ω oscillation Range (χ=54.0, ϕ=0.0)	-60.0 - 120.0^{o}
Exposure Rate	24.0 sec./^{o}
Detector Swing Angle	30.00^{o}
ω oscillation Range (χ=54.0, ϕ=120.0)	-60.0 - 120.0^{o}

Exposure Rate	24.0 sec./^{o}
Detector Swing Angle	30.00^{o}
ω oscillation Range (χ=54.0, ϕ=240.0)	-60.0 - 120.0^{o}
Exposure Rate	24.0 sec./^{o}
Detector Swing Angle	30.00^{o}
Detector Position	50.00 mm
Pixel Size	0.073 mm
$2\theta_{max}$	55.0^{o}
No. of Reflections Measured	Total: 13313<br>Unique: 5746 (R_{int} = 0.0466)<br>Parsons quotients (Flack x parameter):
1898	
Corrections	Lorentz-polarization<br>Absorption<br>(trans. factors: 0.823 - 0.989)

C. Structure Solution and Refinement

Structure Solution	Direct Methods
Refinement	Full-matrix least-squares on F^2
Function Minimized	Σ w $(Fo^2 - Fc^2)^2$
Least Squares Weights	w = 1/ [$\sigma^2(Fo^2)$ + (0.0464 · P)^2 + 0.0000 · P] where P = (Max(Fo^2,0) + 2Fc^2)/3
$2\theta_{max}$ cutoff	54.9^o
Anomalous Dispersion	All non-hydrogen atoms
No. Observations (All reflections)	5746
No. Variables	316
Reflection/Parameter Ratio	18.18
Residuals: R1 (I>2.00σ(I))	0.0450
Residuals: R (All reflections)	0.0571
Residuals: wR2 (All reflections)	0.1003
Goodness of Fit Indicator	1.019
Flack parameter (Parsons' quotients = 1898)	-0.6(8)
Max Shift/Error in Final Cycle	0.000
Maximum peak in Final Diff. Map	0.20 $e^-/Å^3$
Minimum peak in Final Diff. Map	-0.24 $e^-/Å^3$

Table 1. Atomic coordinates and B_{iso}/B_{eq}

atom	x	y	z	B_{eq}
O1	0.37177(15)	0.1144(3)	0.71878(9)	1.76(3)
O2	0.17448(15)	0.9905(3)	0.80976(9)	1.82(3)
N1	0.38245(17)	0.7280(3)	0.81515(10)	1.33(3)
C1	0.4688(2)	0.3738(4)	0.79801(13)	1.40(4)
C2	0.1487(2)	0.7852(4)	0.81954(13)	1.48(4)
C3	0.1044(2)	0.6847(4)	0.74456(13)	1.68(4)
C4	0.4123(2)	0.5734(4)	0.76367(12)	1.25(4)
C5	0.5966(2)	0.5665(4)	0.58938(12)	1.62(4)
C6	0.5517(2)	0.2893(4)	0.67861(13)	1.63(4)
C7	0.0371(2)	0.7744(4)	0.86671(12)	1.49(4)
C8	0.4573(2)	0.2393(4)	0.73185(12)	1.37(4)
C9	0.6697(2)	0.7398(4)	0.59579(14)	2.08(5)
C10	0.5106(2)	0.4995(4)	0.64651(12)	1.43(4)
C11	0.0217(2)	0.6134(4)	0.91228(13)	1.85(4)
C12	-0.1709(2)	0.7583(4)	0.94964(13)	2.08(5)
C13	0.5998(2)	0.4500(4)	0.52606(13)	2.11(5)
C14	0.2744(2)	0.6859(4)	0.85796(12)	1.40(4)
C15	0.6094(2)	0.3704(4)	0.83827(14)	1.75(4)
C16	0.3188(2)	0.7694(4)	0.93579(13)	1.90(4)
C17	0.4926(2)	0.6553(4)	0.70568(13)	1.42(4)
C18	-0.0535(2)	0.9269(4)	0.86271(13)	1.87(4)
C19	0.4891(2)	0.8215(4)	0.86377(13)	1.65(4)
C20	0.6914(2)	0.2746(4)	0.71801(13)	1.90(4)
C21	-0.0814(2)	0.6050(4)	0.95401(14)	2.09(5)
C22	0.6713(3)	0.5078(5)	0.47093(14)	2.48(5)
C23	0.7169(2)	0.3965(4)	0.78903(14)	2.01(5)
C24	-0.1568(2)	0.9179(4)	0.90396(14)	2.23(5)
C25	0.4668(2)	0.7651(4)	0.94199(13)	1.92(4)
C26	0.0653(2)	0.4860(4)	0.74146(14)	2.08(5)
C27	0.7411(3)	0.7978(5)	0.54005(15)	2.58(5)
C28	0.1055(2)	0.7867(5)	0.67846(13)	2.38(5)
C29	0.7416(3)	0.6837(5)	0.47762(15)	2.63(5)
C30	0.0277(3)	0.4982(6)	0.60898(16)	3.28(6)
C31	0.0275(2)	0.3937(5)	0.67416(16)	2.73(5)
C32	0.0672(3)	0.6941(6)	0.61132(16)	3.32(6)

$B_{eq} = 8/3\ \pi^2(U_{11}(aa^*)^2 + U_{22}(bb^*)^2 + U_{33}(cc^*)^2 + 2U_{12}(aa^*bb^*)\cos\gamma + 2U_{13}(aa^*cc^*)\cos\beta + 2U_{23}(bb^*cc^*)\cos\alpha)$

Table 2. Atomic coordinates and B_{iso} involving hydrogen atoms

atom	x	y	z	B_{iso}
H1	0.40942	0.32344	0.83287	1.676
H2	0.23298	1.00283	0.78227	2.189
H4	0.32663	0.53679	0.73528	1.496
H6	0.54074	0.19062	0.63722	1.957
H9	0.67102	0.81975	0.63885	2.501
H10	0.42231	0.48045	0.61818	1.713
H11	0.08248	0.50714	0.91508	2.225
H12	-0.24156	0.75362	0.97792	2.498
H13	0.55198	0.32926	0.52091	2.527
H14	0.26124	0.53893	0.86092	1.683
H15A	0.61820	0.47728	0.87593	2.099
H15B	0.62340	0.24213	0.86480	2.099
H16A	0.28674	0.90679	0.94061	2.275
H16B	0.28738	0.68513	0.97433	2.275
H17A	0.57884	0.69762	0.73036	1.709
H17B	0.44849	0.77354	0.68176	1.709
H18	-0.04487	1.03808	0.83157	2.247
H19A	0.48722	0.96775	0.85745	1.977
H19B	0.57414	0.77100	0.85273	1.977
H20A	0.71110	0.13328	0.72999	2.278
H20B	0.75177	0.31937	0.68353	2.278
H21	-0.09019	0.49431	0.98537	2.512
H22	0.67243	0.42677	0.42830	2.978
H23A	0.72358	0.53886	0.77618	2.413
H23B	0.80094	0.35537	0.81686	2.413
H24	-0.21838	1.02302	0.90065	2.675
H25A	0.50659	0.86304	0.97869	2.300
H25B	0.50145	0.63105	0.95555	2.300
H26	0.06456	0.41308	0.78606	2.494
H27	0.79003	0.91751	0.54516	3.101
H28	0.13287	0.92162	0.67929	2.851
H29	0.78965	0.72489	0.43934	3.157
H30	0.00073	0.43549	0.56282	3.934
H31	0.00145	0.25821	0.67285	3.279
H32	0.06813	0.76610	0.56653	3.986

Table 3. Anisotropic displacement parameters

atom	U_{11}	U_{22}	U_{33}	U_{12}	U_{13}	U_{23}
O1	0.0248(9)	0.0185(10)	0.0242(9)	-0.0037(8)	0.0049(7)	0.0005(8)
O2	0.0244(9)	0.0186(9)	0.0284(10)	0.0019(8)	0.0113(7)	0.0033(8)
N1	0.0154(9)	0.0169(11)	0.0188(10)	0.0002(8)	0.0047(7)	-0.0007(9)
C1	0.0185(11)	0.0165(12)	0.0188(11)	0.0001(10)	0.0054(9)	0.0026(10)
C2	0.0188(11)	0.0175(12)	0.0209(12)	0.0038(10)	0.0059(9)	0.0030(11)
C3	0.0136(10)	0.0297(15)	0.0211(12)	0.0044(10)	0.0050(9)	-0.0003(11)
C4	0.0173(11)	0.0156(12)	0.0152(11)	-0.0000(9)	0.0049(9)	-0.0010(10)
C5	0.0178(11)	0.0266(14)	0.0179(12)	0.0016(10)	0.0056(9)	0.0037(11)
C6	0.0225(12)	0.0182(13)	0.0224(12)	0.0015(10)	0.0071(9)	-0.0030(11)
C7	0.0160(11)	0.0238(13)	0.0167(11)	-0.0004(10)	0.0023(8)	-0.0019(11)
C8	0.0202(11)	0.0127(12)	0.0195(12)	0.0043(10)	0.0032(9)	0.0028(10)
C9	0.0273(13)	0.0276(14)	0.0255(13)	-0.0043(12)	0.0077(10)	0.0001(12)
C10	0.0170(11)	0.0203(12)	0.0179(11)	-0.0011(10)	0.0059(9)	0.0004(11)
C11	0.0180(11)	0.0293(15)	0.0243(13)	0.0030(11)	0.0069(10)	0.0029(12)
C12	0.0136(11)	0.0443(17)	0.0221(12)	-0.0001(12)	0.0057(9)	-0.0042(13)
C13	0.0261(13)	0.0329(16)	0.0213(13)	-0.0013(12)	0.0043(10)	-0.0029(12)
C14	0.0182(11)	0.0185(13)	0.0177(12)	0.0022(10)	0.0065(9)	0.0011(10)
C15	0.0225(12)	0.0204(13)	0.0230(12)	0.0029(10)	0.0009(10)	-0.0012(11)
C16	0.0233(12)	0.0312(15)	0.0181(12)	0.0033(11)	0.0050(9)	0.0011(12)
C17	0.0189(11)	0.0179(13)	0.0180(11)	0.0011(10)	0.0050(9)	0.0019(10)
C18	0.0216(12)	0.0267(15)	0.0231(12)	0.0047(11)	0.0037(9)	0.0019(11)
C19	0.0200(12)	0.0183(13)	0.0245(12)	-0.0008(10)	0.0037(9)	-0.0025(10)
C20	0.0187(11)	0.0268(14)	0.0277(13)	0.0047(11)	0.0070(9)	0.0015(12)
C21	0.0215(12)	0.0365(16)	0.0221(13)	-0.0044(12)	0.0049(10)	0.0039(13)
C22	0.0308(14)	0.0458(18)	0.0189(13)	0.0025(13)	0.0080(10)	-0.0020(13)
C23	0.0170(12)	0.0255(14)	0.0336(14)	0.0015(11)	0.0018(10)	0.0003(13)
C24	0.0191(12)	0.0363(17)	0.0291(14)	0.0094(12)	0.0023(10)	-0.0034(13)
C25	0.0228(12)	0.0290(15)	0.0209(12)	0.0026(11)	0.0026(9)	-0.0048(12)
C26	0.0202(12)	0.0322(15)	0.0276(14)	-0.0014(11)	0.0073(10)	-0.0053(13)
C27	0.0287(13)	0.0365(17)	0.0342(15)	-0.0082(13)	0.0085(11)	0.0056(14)
C28	0.0300(13)	0.0360(17)	0.0240(13)	0.0068(13)	0.0027(10)	0.0018(13)
C29	0.0242(13)	0.052(2)	0.0255(14)	-0.0000(13)	0.0117(11)	0.0074(14)
C30	0.0332(15)	0.062(2)	0.0268(15)	0.0077(16)	-0.0050(12)	-0.0160(16)
C31	0.0190(12)	0.0408(17)	0.0439(17)	0.0002(12)	0.0034(11)	-0.0181(15)
C32	0.0461(17)	0.056(2)	0.0231(14)	0.0122(16)	-0.0003(12)	0.0034(15)

The general temperature factor expression: $\exp(-2\pi^2(a^{*2}U_{11}h^2 + b^{*2}U_{22}k^2 + c^{*2}U_{33}l^2 + 2a^*b^*U_{12}hk + 2a^*c^*U_{13}hl + 2b^*c^*U_{23}kl))$

Table 4. Bond lengths (Å)

atom	atom	distance	atom	atom	distance
O1	C8	1.223(3)	O2	C2	1.421(3)
N1	C4	1.460(3)	N1	C14	1.474(3)
N1	C19	1.467(3)	C1	C4	1.562(3)
C1	C8	1.500(3)	C1	C15	1.546(3)
C2	C3	1.540(3)	C2	C7	1.531(3)
C2	C14	1.550(3)	C3	C26	1.395(4)
C3	C28	1.388(4)	C4	C17	1.531(3)
C5	C9	1.387(4)	C5	C10	1.525(3)
C5	C13	1.399(3)	C6	C8	1.504(3)
C6	C10	1.568(3)	C6	C20	1.536(3)
C7	C11	1.386(4)	C7	C18	1.387(3)
C9	C27	1.388(4)	C10	C17	1.532(3)
C11	C21	1.392(4)	C12	C21	1.384(4)
C12	C24	1.377(4)	C13	C22	1.380(4)
C14	C16	1.539(3)	C15	C23	1.530(4)
C16	C25	1.525(3)	C18	C24	1.388(4)
C19	C25	1.522(3)	C20	C23	1.526(4)
C22	C29	1.387(4)	C26	C31	1.385(4)
C27	C29	1.373(4)	C28	C32	1.384(4)
C30	C31	1.380(4)	C30	C32	1.379(5)

Table 5. Bond lengths involving hydrogens (Å)

atom	atom	distance	atom	atom	distance
O2	H2	0.840	C1	H1	1.000
C4	H4	1.000	C6	H6	1.000
C9	H9	0.950	C10	H10	1.000
C11	H11	0.950	C12	H12	0.950
C13	H13	0.950	C14	H14	1.000
C15	H15A	0.990	C15	H15B	0.990
C16	H16A	0.990	C16	H16B	0.990
C17	H17A	0.990	C17	H17B	0.990
C18	H18	0.950	C19	H19A	0.990
C19	H19B	0.990	C20	H20A	0.990
C20	H20B	0.990	C21	H21	0.950
C22	H22	0.950	C23	H23A	0.990
C23	H23B	0.990	C24	H24	0.950
C25	H25A	0.990	C25	H25B	0.990
C26	H26	0.950	C27	H27	0.950
C28	H28	0.950	C29	H29	0.950
C30	H30	0.950	C31	H31	0.950
C32	H32	0.950			

Table 6. Bond angles (^{O})

atom	atom	atom	angle	atom	atom	atom	angle
C4	N1	C14	116.28(18)	C4	N1	C19	119.11(18)
C14	N1	C19	109.22(17)	C4	C1	C8	102.08(18)
C4	C1	C15	119.0(2)	C8	C1	C15	110.14(19)
O2	C2	C3	110.75(19)	O2	C2	C7	106.56(19)
O2	C2	C14	108.29(17)	C3	C2	C7	108.46(18)
C3	C2	C14	110.40(19)	C7	C2	C14	112.32(19)
C2	C3	C26	120.5(2)	C2	C3	C28	121.2(2)
C26	C3	C28	118.3(2)	N1	C4	C1	117.00(18)
N1	C4	C17	111.54(19)	C1	C4	C17	112.08(19)
C9	C5	C10	123.6(2)	C9	C5	C13	117.9(2)
C10	C5	C13	118.5(2)	C8	C6	C10	105.88(19)
C8	C6	C20	109.81(19)	C10	C6	C20	115.3(2)
C2	C7	C11	121.9(2)	C2	C7	C18	119.5(2)
C11	C7	C18	118.6(2)	O1	C8	C1	123.4(2)
O1	C8	C6	123.0(2)	C1	C8	C6	113.15(19)
C5	C9	C27	120.8(2)	C5	C10	C6	111.45(19)
C5	C10	C17	114.5(2)	C6	C10	C17	113.95(18)
C7	C11	C21	121.0(2)	C21	C12	C24	119.5(2)
C5	C13	C22	121.0(3)	N1	C14	C2	109.38(18)
N1	C14	C16	105.08(17)	C2	C14	C16	113.1(2)
C1	C15	C23	115.8(2)	C14	C16	C25	103.87(19)
C4	C17	C10	111.52(19)	C7	C18	C24	120.4(2)
N1	C19	C25	105.30(19)	C6	C20	C23	114.3(2)
C11	C21	C12	119.8(3)	C13	C22	C29	120.2(3)
C15	C23	C20	112.1(2)	C12	C24	C18	120.8(2)
C16	C25	C19	101.57(18)	C3	C26	C31	120.8(3)
C9	C27	C29	120.6(3)	C3	C28	C32	120.7(3)
C22	C29	C27	119.4(3)	C31	C30	C32	119.5(3)
C26	C31	C30	120.2(3)	C28	C32	C30	120.5(3)

Table 7. Bond angles involving hydrogens (^{O})

atom	atom	atom	angle	atom	atom	atom	angle
C2	O2	H2	109.5	C4	C1	H1	108.4
C8	C1	H1	108.4	C15	C1	H1	108.4
N1	C4	H4	105.0	C1	C4	H4	105.0
C17	C4	H4	105.0	C8	C6	H6	108.5
C10	C6	H6	108.5	C20	C6	H6	108.5
C5	C9	H9	119.6	C27	C9	H9	119.6
C5	C10	H10	105.3	C6	C10	H10	105.3
C17	C10	H10	105.3	C7	C11	H11	119.5
C21	C11	H11	119.5	C21	C12	H12	120.3
C24	C12	H12	120.3	C5	C13	H13	119.5
C22	C13	H13	119.5	N1	C14	H14	109.7
C2	C14	H14	109.7	C16	C14	H14	109.7
C1	C15	H15A	108.3	C1	C15	H15B	108.3
C23	C15	H15A	108.3	C23	C15	H15B	108.3
H15A	C15	H15B	107.4	C14	C16	H16A	111.0
C14	C16	H16B	111.0	C25	C16	H16A	111.0
C25	C16	H16B	111.0	H16A	C16	H16B	109.0
C4	C17	H17A	109.3	C4	C17	H17B	109.3
C10	C17	H17A	109.3	C10	C17	H17B	109.3
H17A	C17	H17B	108.0	C7	C18	H18	119.8
C24	C18	H18	119.8	N1	C19	H19A	110.7
N1	C19	H19B	110.7	C25	C19	H19A	110.7
C25	C19	H19B	110.7	H19A	C19	H19B	108.8
C6	C20	H20A	108.7	C6	C20	H20B	108.7
C23	C20	H20A	108.7	C23	C20	H20B	108.7
H20A	C20	H20B	107.6	C11	C21	H21	120.1
C12	C21	H21	120.1	C13	C22	H22	119.9
C29	C22	H22	119.9	C15	C23	H23A	109.2
C15	C23	H23B	109.2	C20	C23	H23A	109.2
C20	C23	H23B	109.2	H23A	C23	H23B	107.9
C12	C24	H24	119.6	C18	C24	H24	119.6
C16	C25	H25A	111.5	C16	C25	H25B	111.5
C19	C25	H25A	111.5	C19	C25	H25B	111.5
H25A	C25	H25B	109.3	C3	C26	H26	119.6
C31	C26	H26	119.6	C9	C27	H27	119.7
C29	C27	H27	119.7	C3	C28	H28	119.6
C32	C28	H28	119.6	C22	C29	H29	120.3

Table 7. Bond angles involving hydrogens (^{o}) (continued)

atom	atom	atom	angle	atom	atom	atom	angle
C27	C29	H29	120.3	C31	C30	H30	120.2
C32	C30	H30	120.2	C26	C31	H31	119.9
C30	C31	H31	119.9	C28	C32	H32	119.8
C30	C32	H32	119.8				

Table 8. Torsion Angles(°)
(Those having bond angles > 160 or < 20 degrees are excluded.)

atom1	atom2	atom3	atom4	angle	atom1	atom2	atom3	atom4	angle
C4	N1	C14	C2	-97.2(2)	C4	N1	C14	C16	141.03(16)
C14	N1	C4	C1	-67.5(2)	C14	N1	C4	C17	161.63(15)
C4	N1	C19	C25	-115.81(19)	C19	N1	C4	C1	66.6(3)
C19	N1	C4	C17	-64.3(2)	C14	N1	C19	C25	21.1(2)
C19	N1	C14	C2	124.49(17)	C19	N1	C14	C16	2.7(2)
C4	C1	C8	O1	-102.1(2)	C4	C1	C8	C6	71.1(2)
C8	C1	C4	N1	167.16(17)	C8	C1	C4	C17	-62.2(2)
C4	C1	C15	C23	-69.1(3)	C15	C1	C4	N1	-71.4(3)
C15	C1	C4	C17	59.3(3)	C8	C1	C15	C23	48.1(3)
C15	C1	C8	O1	130.5(2)	C15	C1	C8	C6	-56.2(2)
O2	C2	C3	C26	-175.58(16)	O2	C2	C3	C28	6.1(3)
O2	C2	C7	C11	-152.34(17)	O2	C2	C7	C18	29.3(2)
O2	C2	C14	N1	-54.1(2)	O2	C2	C14	C16	62.7(2)
C3	C2	C7	C11	88.4(2)	C3	C2	C7	C18	-90.0(2)
C7	C2	C3	C26	-59.0(3)	C7	C2	C3	C28	122.7(2)
C3	C2	C14	N1	67.3(2)	C3	C2	C14	C16	-175.91(17)
C14	C2	C3	C26	64.5(3)	C14	C2	C3	C28	-113.8(2)
C7	C2	C14	N1	-171.49(17)	C7	C2	C14	C16	-54.7(2)
C14	C2	C7	C11	-33.9(3)	C14	C2	C7	C18	147.74(18)
C2	C3	C26	C31	-178.87(18)	C2	C3	C28	C32	179.11(19)
C26	C3	C28	C32	0.8(3)	C28	C3	C26	C31	-0.5(3)
N1	C4	C17	C10	-173.44(14)	C1	C4	C17	C10	53.2(2)
C9	C5	C10	C6	-121.4(2)	C9	C5	C10	C17	9.7(3)
C10	C5	C9	C27	-176.72(18)	C9	C5	C13	C22	-1.3(3)
C13	C5	C9	C27	1.6(3)	C10	C5	C13	C22	177.10(18)
C13	C5	C10	C6	60.3(2)	C13	C5	C10	C17	-168.58(18)
C8	C6	C10	C5	-178.27(15)	C8	C6	C10	C17	50.3(2)
C10	C6	C8	O1	107.4(2)	C10	C6	C8	C1	-65.8(2)
C8	C6	C20	C23	-53.5(3)	C20	C6	C8	O1	-127.4(2)
C20	C6	C8	C1	59.3(2)	C10	C6	C20	C23	66.0(3)
C20	C6	C10	C5	60.1(2)	C20	C6	C10	C17	-71.3(2)
C2	C7	C11	C21	-179.14(18)	C2	C7	C18	C24	178.76(17)
C11	C7	C18	C24	0.4(3)	C18	C7	C11	C21	-0.8(3)
C5	C9	C27	C29	-0.6(4)	C5	C10	C17	C4	-176.48(15)
C6	C10	C17	C4	-46.6(2)	C7	C11	C21	C12	0.6(3)
C21	C12	C24	C18	-0.3(4)	C24	C12	C21	C11	-0.1(3)
C5	C13	C22	C29	-0.0(4)	N1	C14	C16	C25	-25.4(2)

Table 8. Torsion angles (°) (continued)

atom1	atom2	atom3	atom4	angle	atom1	atom2	atom3	atom4	angle
C2	C14	C16	C25	-144.63(18)	C1	C15	C23	C20	-43.3(3)
C14	C16	C25	C19	37.3(2)	C7	C18	C24	C12	0.2(3)
N1	C19	C25	C16	-36.1(2)	C6	C20	C23	C15	45.8(3)
C13	C22	C29	C27	1.1(4)	C3	C26	C31	C30	-0.3(4)
C9	C27	C29	C22	-0.8(4)	C3	C28	C32	C30	-0.3(4)
C31	C30	C32	C28	-0.5(4)	C32	C30	C31	C26	0.8(4)

Table 9. Possible hydrogen bonds

Donor	H	Acceptor	D...A	D-H	H...A	D-H...A	
O2	H2	O1[1]	2.919(2)	0.84	2.10	164.74	
O2	H2	N1	2.780(2)	0.84	2.44	105.35	intramol.

Symmetry Operators:

(1) X,Y+1,Z

Table 10. Intramolecular contacts less than 3.60 Å

atom	atom	distance	atom	atom	distance
O1	C4	3.206(3)	O1	C10	3.318(3)
O1	C15	3.521(3)	O1	C20	3.490(3)
O2	N1	2.780(2)	O2	C16	2.973(3)
O2	C18	2.703(3)	O2	C19	3.478(3)
O2	C28	2.770(3)	N1	C3	3.018(3)
N1	C15	3.354(3)	N1	C28	3.573(3)
C1	C10	2.973(3)	C1	C14	3.200(3)
C1	C19	3.237(3)	C1	C20	2.964(3)
C2	C4	3.358(3)	C2	C19	3.530(3)
C3	C4	3.257(3)	C3	C11	3.315(3)
C3	C18	3.300(4)	C3	C30	2.793(4)
C4	C6	2.954(3)	C4	C23	3.353(3)
C4	C25	3.474(3)	C5	C20	3.117(3)
C5	C29	2.798(4)	C6	C13	3.083(4)
C6	C15	2.944(3)	C7	C12	2.794(3)
C7	C16	3.030(3)	C7	C26	3.038(4)
C7	C28	3.593(3)	C8	C17	2.869(3)
C8	C23	2.956(3)	C9	C17	2.944(4)
C9	C22	2.761(4)	C10	C23	3.220(3)
C11	C14	2.962(3)	C11	C16	3.233(3)
C11	C24	2.752(4)	C11	C26	3.316(4)
C13	C27	2.754(4)	C14	C26	3.135(3)
C14	C28	3.570(3)	C15	C17	3.197(3)
C15	C19	3.336(4)	C17	C19	3.095(3)
C17	C20	3.278(4)	C17	C23	3.136(3)
C18	C21	2.768(4)	C26	C32	2.756(4)
C28	C31	2.762(4)			

Table 11. Intramolecular contacts less than 3.60 Å involving hydrogens

atom	atom	distance	atom	atom	distance
O1	H1	2.498	O1	H4	2.900
O1	H6	2.495	O1	H10	3.153
O1	H15B	3.583	O1	H20A	3.504
O2	H14	3.268	O2	H16A	2.577
O2	H18	2.383	O2	H19A	3.254
O2	H28	2.406	N1	H1	2.749
N1	H2	2.437	N1	H15A	3.058
N1	H16A	2.872	N1	H16B	3.196
N1	H17A	2.721	N1	H17B	2.628
N1	H25A	3.220	N1	H25B	2.771
N1	H28	3.588	C1	H6	3.351
C1	H10	3.328	C1	H14	2.798
C1	H17A	2.816	C1	H17B	3.412
C1	H19B	3.007	C1	H20A	3.360
C1	H23A	2.942	C1	H23B	3.422
C1	H25B	3.331	C2	H4	3.049
C2	H11	2.704	C2	H16A	2.602
C2	H16B	3.071	C2	H18	2.663
C2	H26	2.694	C2	H28	2.700
C3	H2	2.567	C3	H4	2.538
C3	H11	3.364	C3	H14	2.685
C3	H18	3.347	C3	H31	3.273
C3	H32	3.264	C4	H2	3.476
C4	H10	2.739	C4	H14	2.525
C4	H15A	2.835	C4	H15B	3.480
C4	H19A	3.196	C4	H19B	2.552
C4	H23A	3.218	C4	H25B	3.521
C5	H6	2.760	C5	H17A	2.745
C5	H17B	2.794	C5	H20B	2.754
C5	H22	3.274	C5	H23A	3.492
C5	H27	3.266	C6	H1	3.349
C6	H4	3.150	C6	H13	2.887
C6	H15B	3.393	C6	H17A	2.905
C6	H17B	3.430	C6	H23A	2.887
C6	H23B	3.398	C7	H2	3.110
C7	H14	2.827	C7	H16A	2.901
C7	H16B	3.104	C7	H21	3.273

Table 11. Intramolecular contacts less than 3.60 Å involving hydrogens (continued)

atom	atom	distance	atom	atom	distance
C7	H24	3.261	C7	H26	2.872
C8	H4	2.422	C8	H10	2.619
C8	H15A	3.333	C8	H15B	2.786
C8	H17A	3.331	C8	H20A	2.734
C8	H20B	3.333	C8	H23A	3.431
C9	H10	3.174	C9	H13	3.247
C9	H17A	2.754	C9	H17B	2.952
C9	H20B	3.304	C9	H23A	3.531
C9	H29	3.257	C10	H4	2.670
C10	H9	2.737	C10	H13	2.644
C10	H20A	3.448	C10	H20B	2.784
C10	H23A	3.031	C11	H12	3.261
C11	H14	2.813	C11	H16A	3.370
C11	H16B	2.882	C11	H18	3.245
C11	H26	2.751	C12	H11	3.255
C12	H18	3.257	C13	H6	2.801
C13	H9	3.249	C13	H10	2.657
C13	H20B	3.213	C13	H29	3.256
C14	H1	2.877	C14	H2	2.545
C14	H4	2.571	C14	H11	2.653
C14	H19A	2.911	C14	H19B	3.177
C14	H25A	3.270	C14	H25B	2.787
C14	H26	3.014	C15	H4	3.459
C15	H17A	2.941	C15	H19B	2.736
C15	H20A	2.844	C15	H20B	3.367
C15	H25B	3.085	C16	H2	3.231
C16	H11	3.005	C16	H19A	2.742
C16	H19B	3.218	C17	H1	3.408
C17	H6	3.425	C17	H9	2.592
C17	H15A	3.422	C17	H19A	3.480
C17	H19B	2.813	C17	H20B	3.575
C17	H23A	2.686	C18	H2	3.519
C18	H11	3.243	C18	H12	3.262
C19	H1	3.478	C19	H2	3.121
C19	H4	3.312	C19	H14	3.029
C19	H15A	2.669	C19	H16A	2.733
C19	H16B	3.222	C19	H17A	2.839

Table 11. Intramolecular contacts less than 3.60 Å involving hydrogens (continued)

atom	atom	distance	atom	atom	distance
C19	H17B	3.305	C20	H10	3.422
C20	H15A	3.359	C20	H15B	2.862
C20	H17A	3.095	C21	H24	3.242
C22	H27	3.241	C23	H1	3.425
C23	H6	3.406	C23	H17A	2.627
C23	H19B	3.214	C24	H21	3.244
C25	H1	3.581	C25	H14	2.869
C25	H15A	2.856	C26	H4	2.751
C26	H11	3.149	C26	H14	2.803
C26	H28	3.250	C26	H30	3.255
C27	H22	3.241	C28	H2	2.606
C28	H4	2.924	C28	H17B	3.554
C28	H26	3.249	C28	H30	3.257
C29	H9	3.250	C29	H13	3.253
C30	H26	3.251	C30	H28	3.252
C31	H4	3.303	C31	H32	3.242
C32	H4	3.449	C32	H31	3.242
H1	H4	2.359	H1	H14	2.221
H1	H15A	2.437	H1	H15B	2.288
H1	H19B	3.457	H1	H25B	3.104
H2	H4	3.423	H2	H14	3.429
H2	H16A	2.939	H2	H17B	3.435
H2	H18	3.139	H2	H19A	2.821
H2	H28	2.096	H4	H10	2.497
H4	H14	2.471	H4	H17A	2.845
H4	H17B	2.325	H4	H19B	3.498
H4	H26	3.099	H4	H28	3.354
H6	H10	2.306	H6	H13	2.332
H6	H20A	2.315	H6	H20B	2.404
H9	H10	3.429	H9	H17A	2.187
H9	H17B	2.552	H9	H20B	3.537
H9	H23A	3.126	H9	H27	2.328
H10	H13	2.570	H10	H17A	2.844
H10	H17B	2.284	H11	H14	2.222
H11	H16A	3.417	H11	H16B	2.555
H11	H21	2.336	H11	H26	2.419
H12	H21	2.339	H12	H24	2.326

Table 11. Intramolecular contacts less than 3.60 Å involving hydrogens (continued)

atom	atom	distance	atom	atom	distance
H13	H20B	3.390	H13	H22	2.321
H14	H16A	2.863	H14	H16B	2.273
H14	H25B	2.908	H14	H26	2.454
H15A	H17A	3.020	H15A	H19A	3.566
H15A	H19B	2.059	H15A	H23A	2.278
H15A	H23B	2.441	H15A	H25A	3.484
H15A	H25B	2.258	H15B	H19B	3.595
H15B	H20A	2.825	H15B	H23A	2.847
H15B	H23B	2.271	H15B	H25B	3.423
H16A	H19A	2.759	H16A	H25A	2.316
H16A	H25B	2.885	H16B	H25A	2.563
H16B	H25B	2.320	H17A	H19A	3.184
H17A	H19B	2.291	H17A	H20B	3.289
H17A	H23A	1.941	H17A	H23B	3.486
H17B	H19A	3.433	H17B	H19B	3.218
H17B	H23A	3.511	H17B	H28	3.419
H18	H24	2.330	H19A	H25A	2.303
H19A	H25B	2.877	H19B	H23A	2.711
H19B	H25A	2.562	H19B	H25B	2.309
H20A	H23A	2.852	H20A	H23B	2.285
H20B	H23A	2.290	H20B	H23B	2.429
H22	H29	2.340	H26	H31	2.328
H27	H29	2.323	H28	H32	2.326
H30	H31	2.332	H30	H32	2.329

Table 12. Intermolecular contacts less than 3.60 Å

atom	atom	distance	atom	atom	distance
O1	O2[1]	2.919(2)	O1	N1[1]	3.130(3)
O1	C17[1]	3.352(3)	O1	C19[1]	3.393(3)
O1	C22[2]	3.502(3)	O1	C28[1]	3.536(3)
O2	O1[3]	2.919(2)	N1	O1[3]	3.130(3)
C17	O1[3]	3.352(3)	C19	O1[3]	3.393(3)
C21	C24[4]	3.568(3)	C22	O1[5]	3.502(3)
C24	C21[6]	3.568(3)	C27	C32[7]	3.534(4)
C28	O1[3]	3.536(3)	C32	C27[8]	3.534(4)

Symmetry Operators:

(1) X,Y-1,Z
(2) -X+1,Y+1/2-1,-Z+1
(3) X,Y+1,Z
(4) -X,Y+1/2-1,-Z+2
(5) -X+1,Y+1/2,-Z+1
(6) -X,Y+1/2,-Z+2
(7) X+1,Y,Z
(8) X-1,Y,Z

Table 13. Intermolecular contacts less than 3.60 Å involving hydrogens

atom	atom	distance	atom	atom	distance
O1	H2[1]	2.100	O1	H17A[1]	3.521
O1	H17B[1]	2.546	O1	H19A[1]	2.830
O1	H22[2]	2.944	O1	H28[1]	2.808
O1	H29[2]	3.226	O2	H1[3]	3.297
O2	H26[3]	3.072	O2	H31[3]	3.396
C1	H2[1]	3.481	C1	H19A[1]	2.934
C5	H13[4]	2.956	C6	H9[1]	3.502
C7	H21[5]	3.061	C8	H2[1]	3.058
C8	H17B[1]	3.260	C8	H19A[1]	2.913
C9	H6[3]	3.437	C9	H13[4]	2.985
C9	H20A[3]	3.591	C11	H21[5]	3.194
C11	H23B[6]	3.202	C12	H11[5]	3.022
C12	H15A[6]	3.067	C12	H16A[7]	3.414
C12	H16B[5]	3.472	C12	H19B[6]	2.986
C12	H21[5]	3.230	C12	H23A[6]	3.532
C12	H25A[6]	3.527	C12	H25B[6]	3.522
C13	H6[4]	3.532	C13	H13[4]	3.061
C15	H19A[1]	3.029	C15	H24[8]	3.068
C16	H21[5]	3.297	C16	H25A[9]	3.528
C16	H25B[10]	3.515	C17	H22[4]	3.338
C18	H20A[11]	3.495	C18	H21[5]	3.004
C18	H23B[11]	3.310	C19	H1[3]	3.503
C19	H12[12]	3.293	C19	H15B[3]	3.152
C19	H24[12]	3.313	C20	H9[1]	3.376
C20	H18[8]	3.583	C20	H31[12]	3.425
C21	H15A[6]	3.367	C21	H16A[7]	3.327
C21	H21[5]	3.276	C21	H23B[6]	3.125
C22	H6[4]	3.019	C22	H13[4]	3.187
C22	H17B[2]	3.297	C22	H32[2]	3.301
C23	H18[8]	3.468	C23	H24[8]	3.247
C24	H11[5]	3.342	C24	H15B[11]	3.170
C24	H16B[5]	3.281	C24	H19B[6]	2.996
C24	H20A[11]	3.592	C24	H21[5]	3.092
C24	H23A[6]	3.565	C24	H23B[11]	3.344
C25	H12[12]	3.015	C25	H25A[9]	3.061
C25	H25B[10]	3.081	C26	H20B[6]	3.474
C26	H23B[6]	3.342	C27	H10[4]	3.381

Table 13. Intermolecular contacts less than 3.60 Å involving hydrogens (continued)

atom	atom	distance	atom	atom	distance
C27	H13^{4}	3.108	C27	H30^{4}	3.586
C27	H32^{12}	3.373	C28	H22^{4}	3.339
C28	H31^{3}	3.347	C29	H6^{4}	3.373
C29	H10^{4}	3.026	C29	H13^{4}	3.204
C29	H30^{12}	3.369	C29	H30^{4}	3.328
C29	H32^{2}	3.584	C30	H20B^{6}	3.536
C30	H29^{2}	2.860	C30	H32^{13}	3.582
C31	H20B^{6}	2.933	C31	H28^{1}	3.355
C31	H29^{2}	3.192	C32	H22^{4}	3.283
C32	H27^{6}	3.330	C32	H30^{14}	3.553
H1	O2^{1}	3.297	H1	C19^{1}	3.503
H1	H2^{1}	2.901	H1	H19A^{1}	2.545
H1	H25A^{9}	3.442	H2	O1^{3}	2.100
H2	C1^{3}	3.481	H2	C8^{3}	3.058
H2	H1^{3}	2.901	H2	H26^{3}	3.271
H2	H31^{3}	3.385	H6	C9^{1}	3.437
H6	C13^{2}	3.532	H6	C22^{2}	3.019
H6	C29^{2}	3.373	H6	H9^{1}	2.835
H6	H17B^{1}	3.105	H6	H22^{2}	2.964
H6	H29^{2}	3.538	H9	C6^{3}	3.502
H9	C20^{3}	3.376	H9	H6^{3}	2.835
H9	H13^{4}	3.477	H9	H20A^{3}	2.683
H9	H20B^{3}	3.532	H10	C27^{2}	3.381
H10	C29^{2}	3.026	H10	H13^{4}	3.489
H10	H22^{4}	3.236	H10	H27^{2}	3.492
H10	H29^{2}	2.880	H11	C12^{7}	3.022
H11	C24^{7}	3.342	H11	H12^{7}	2.932
H11	H23B^{6}	3.381	H11	H24^{7}	3.471
H12	C19^{6}	3.293	H12	C25^{6}	3.015
H12	H11^{5}	2.932	H12	H14^{5}	3.536
H12	H15A^{6}	2.885	H12	H16A^{7}	2.836
H12	H16B^{5}	3.084	H12	H19B^{6}	2.783
H12	H25A^{6}	2.718	H12	H25B^{6}	2.771
H13	C5^{2}	2.956	H13	C9^{2}	2.985
H13	C13^{2}	3.061	H13	C22^{2}	3.187
H13	C27^{2}	3.108	H13	C29^{2}	3.204
H13	H9^{2}	3.477	H13	H10^{2}	3.489

Table 13. Intermolecular contacts less than 3.60 Å involving hydrogens (continued)

atom	atom	distance	atom	atom	distance
H13	H13^{2}	3.583	H13	H13^{4}	3.583
H14	H12^{7}	3.536	H15A	C12^{12}	3.067
H15A	C21^{12}	3.367	H15A	H12^{12}	2.885
H15A	H16A^{9}	3.394	H15A	H16B^{9}	3.402
H15A	H21^{12}	3.404	H15A	H24^{8}	3.495
H15A	H25A^{9}	3.190	H15B	C19^{1}	3.152
H15B	C24^{8}	3.170	H15B	H16B^{9}	2.982
H15B	H19A^{1}	2.317	H15B	H19B^{1}	3.212
H15B	H24^{8}	2.240	H15B	H25A^{1}	3.596
H15B	H25A^{9}	3.408	H16A	C12^{5}	3.414
H16A	C21^{5}	3.327	H16A	H12^{5}	2.836
H16A	H15A^{10}	3.394	H16A	H21^{5}	2.654
H16A	H25B^{10}	3.100	H16B	C12^{7}	3.472
H16B	C24^{7}	3.281	H16B	H12^{7}	3.084
H16B	H15A^{10}	3.402	H16B	H15B^{10}	2.982
H16B	H21^{5}	3.073	H16B	H24^{7}	2.705
H16B	H25A^{9}	3.086	H17A	O1^{3}	3.521
H17A	H20A^{3}	3.235	H17B	O1^{3}	2.546
H17B	C8^{3}	3.260	H17B	C22^{4}	3.297
H17B	H6^{3}	3.105	H17B	H22^{4}	2.451
H18	C20^{11}	3.583	H18	C23^{11}	3.468
H18	H20A^{11}	3.006	H18	H21^{5}	3.459
H18	H23B^{11}	2.660	H18	H26^{3}	2.929
H18	H31^{3}	3.339	H19A	O1^{3}	2.830
H19A	C1^{3}	2.934	H19A	C8^{3}	2.913
H19A	C15^{3}	3.029	H19A	H1^{3}	2.545
H19A	H15B^{3}	2.317	H19A	H24^{12}	3.078
H19A	H25B^{10}	3.568	H19B	C12^{12}	2.986
H19B	C24^{12}	2.996	H19B	H12^{12}	2.783
H19B	H15B^{3}	3.212	H19B	H24^{12}	2.790
H20A	C9^{1}	3.591	H20A	C18^{8}	3.495
H20A	C24^{8}	3.592	H20A	H9^{1}	2.683
H20A	H17A^{1}	3.235	H20A	H18^{8}	3.006
H20A	H24^{8}	3.189	H20A	H31^{12}	3.419
H20B	C26^{12}	3.474	H20B	C30^{12}	3.536
H20B	C31^{12}	2.933	H20B	H9^{1}	3.532
H20B	H26^{12}	3.586	H20B	H31^{12}	2.656

Table 13. Intermolecular contacts less than 3.60 Å involving hydrogens (continued)

atom	atom	distance	atom	atom	distance
H21	C7^{7}	3.061	H21	C11^{7}	3.194
H21	C12^{7}	3.230	H21	C16^{7}	3.297
H21	C18^{7}	3.004	H21	C21^{7}	3.276
H21	C24^{7}	3.092	H21	H15A^{6}	3.404
H21	H16A^{7}	2.654	H21	H16B^{7}	3.073
H21	H18^{7}	3.459	H21	H23B^{6}	3.268
H21	H24^{7}	3.590	H22	O1^{4}	2.944
H22	C17^{2}	3.338	H22	C28^{2}	3.339
H22	C32^{2}	3.283	H22	H6^{4}	2.964
H22	H10^{2}	3.236	H22	H17B^{2}	2.451
H22	H28^{2}	2.993	H22	H32^{2}	2.892
H23A	C12^{12}	3.532	H23A	C24^{12}	3.565
H23B	C11^{12}	3.202	H23B	C18^{8}	3.310
H23B	C21^{12}	3.125	H23B	C24^{8}	3.344
H23B	C26^{12}	3.342	H23B	H11^{12}	3.381
H23B	H18^{8}	2.660	H23B	H21^{12}	3.268
H23B	H24^{8}	2.729	H23B	H26^{12}	2.890
H24	C15^{11}	3.068	H24	C19^{6}	3.313
H24	C23^{11}	3.247	H24	H11^{5}	3.471
H24	H15A^{11}	3.495	H24	H15B^{11}	2.240
H24	H16B^{5}	2.705	H24	H19A^{6}	3.078
H24	H19B^{6}	2.790	H24	H20A^{11}	3.189
H24	H21^{5}	3.590	H24	H23B^{11}	2.729
H24	H25A^{6}	3.519	H25A	C12^{12}	3.527
H25A	C16^{10}	3.528	H25A	C25^{10}	3.061
H25A	H1^{10}	3.442	H25A	H12^{12}	2.718
H25A	H15A^{10}	3.190	H25A	H15B^{3}	3.596
H25A	H15B^{10}	3.408	H25A	H16B^{10}	3.086
H25A	H24^{12}	3.519	H25A	H25A^{9}	3.457
H25A	H25A^{10}	3.457	H25A	H25B^{10}	2.172
H25B	C12^{12}	3.522	H25B	C16^{9}	3.515
H25B	C25^{9}	3.081	H25B	H12^{12}	2.771
H25B	H16A^{9}	3.100	H25B	H19A^{9}	3.568
H25B	H25A^{9}	2.172	H26	O2^{1}	3.072
H26	H2^{1}	3.271	H26	H18^{1}	2.929
H26	H20B^{6}	3.586	H26	H23B^{6}	2.890
H27	C32^{12}	3.330	H27	H10^{4}	3.492

Table 13. Intermolecular contacts less than 3.60 Å involving hydrogens (continued)

atom	atom	distance	atom	atom	distance
H27	H30^{4}	3.117	H27	H32^{12}	3.038
H27	H32^{4}	3.547	H28	O1^{3}	2.808
H28	C31^{3}	3.355	H28	H22^{4}	2.993
H28	H29^{4}	3.151	H28	H31^{3}	2.637
H29	O1^{4}	3.226	H29	C30^{4}	2.860
H29	C31^{4}	3.192	H29	H6^{4}	3.538
H29	H10^{4}	2.880	H29	H28^{2}	3.151
H29	H30^{12}	3.517	H29	H30^{4}	2.601
H29	H31^{4}	3.175	H29	H32^{12}	3.474
H29	H32^{2}	3.428	H30	C27^{2}	3.586
H30	C29^{6}	3.369	H30	C29^{2}	3.328
H30	C32^{13}	3.553	H30	H27^{2}	3.117
H30	H29^{6}	3.517	H30	H29^{2}	2.601
H30	H32^{13}	2.632	H31	O2^{1}	3.396
H31	C20^{6}	3.425	H31	C28^{1}	3.347
H31	H2^{1}	3.385	H31	H18^{1}	3.339
H31	H20A^{6}	3.419	H31	H20B^{6}	2.656
H31	H28^{1}	2.637	H31	H29^{2}	3.175
H32	C22^{4}	3.301	H32	C27^{6}	3.373
H32	C29^{4}	3.584	H32	C30^{14}	3.582
H32	H22^{4}	2.892	H32	H27^{6}	3.038
H32	H27^{2}	3.547	H32	H29^{6}	3.474
H32	H29^{4}	3.428	H32	H30^{14}	2.632

Symmetry Operators:

(1) X,Y-1,Z
(2) -X+1,Y+1/2-1,-Z+1
(3) X,Y+1,Z
(4) -X+1,Y+1/2,-Z+1
(5) -X,Y+1/2,-Z+2
(6) X-1,Y,Z
(7) -X,Y+1/2-1,-Z+2
(8) X+1,Y-1,Z
(9) -X+1,Y+1/2-1,-Z+2
(10) -X+1,Y+1/2,-Z+2
(11) X-1,Y+1,Z
(12) X+1,Y,Z
(13) -X,Y+1/2-1,-Z+1
(14) -X,Y+1/2,-Z+1
